# *CEBPB* Expression in Tumor Cells Drives Immune Evasion in Colorectal Cancer via *CTLA4* Up-regulation in T Cells

**DOI:** 10.34133/cancomm.0013

**Published:** 2026-02-24

**Authors:** Hye Jeong Yun, Chan Ho Park, Dahye Yun, Hye-Ri Shin, Jeong Dong Lee, Changhee Park, Kiyeon Kim, Heejun Shim, Hyejin Sim, Se Min Kim, Min Jung Kim, Ji Won Park, Seung-Bum Ryoo, Yoojoo Lim, Seung-Yong Jeong, Kyu Joo Park, Tae-You Kim, Junil Kim, Jae-Kyung Won, Sae-Won Han

**Affiliations:** ^1^ Cancer Research Institute, Seoul National University College of Medicine, Seoul, Korea.; ^2^ Department of Bioinformatics, Soongsil University, Seoul, Korea.; ^3^ Department of Pathology, Seoul National University Hospital, Seoul National University College of Medicine, Seoul, Korea.; ^4^ Department of Internal Medicine, Seoul National University Hospital, Seoul, Korea; ^5^ Department of Surgery, Seoul National University Hospital, Seoul, Korea.; ^6^ Lunit Inc., Seoul, Korea.; ^7^ School of Systems Biomedical Science, Soongsil University, Seoul, Korea.

## Abstract

**Background:** Immune checkpoint inhibitors are ineffective in the majority of colorectal cancers (CRCs) that are microsatellite stable. However, the underlying reasons for their unresponsiveness and mechanisms of immune evasion are poorly understood. In the present study, we aimed to elucidate the mechanisms underlying the immune evasion driven by CRC cells. **Methods:** We performed single-cell RNA sequencing of tumor tissues from 30 CRC patients and syngeneic mice implanted with transformation-related protein 53 (*Trp53*) knockout CT26 cells. Gene expression and correlations of individual tumor microenvironment (TME) components were analyzed, and their functional significance was investigated using syngeneic mouse models and cell line co-culture experiments. **Results:** CCAAT enhancer-binding protein beta (*CEBPB*) expression was increased in tumor protein 53 (*TP53*)-mutated CRCs. We confirmed that wild-type *TP53* negatively regulated *CEBPB* expression in CRC cell lines. *CEBPB* expression was associated with decreased intratumoral T cell infiltration and negatively impacted survival in CRC patients. In the intercellular correlation analysis of gene expression, tumor epithelial cell *CEBPB* expression was significantly correlated with cytotoxic T-lymphocyte associated protein 4 (*CTLA4*) expression in T cells, especially in regulatory and exhausted T cells. *Cebpb* overexpression promoted tumor growth in the immunocompetent syngeneic mouse models, which was accompanied by increased CTLA-4 expression in tumor-infiltrating CD4^+^ T cells. In vitro co-culture experiments also showed that tumor cell *CEBPB* overexpression increased *CTLA4* in T cells. **Conclusions:** Tumor cell *CEBPB* expression, up-regulated by *TP53* mutation, can increase *CTLA4* expression in T cells and negatively affect patient outcomes. These findings suggested a central role of tumor cell *CEBPB* in shaping an immunosuppressive TME.

## Background

Immunotherapy using immune checkpoint blockade (ICB) has opened a new era in cancer treatment. It has substantially changed the treatment landscape of many cancers, including melanoma, lung cancer, and renal cell carcinoma [1]. However, treatment with anti-programmed cell death protein 1 (PD-1) or programmed cell death ligand 1 (PD-L1) has failed to show a response in the majority of colorectal cancers (CRCs) that are microsatellite stable (MSS), and the efficacy was limited to a subset of patients with microsatellite instability-high (MSI-H) [2–4]. MSI-H tumors are more immunogenic compared to MSS CRC due to their high tumor mutational burden (TMB) and neoantigen load [5]. However, MSS CRC is less responsive to ICB than other cancers with similar TMB levels, suggesting the involvement of additional resistance mechanisms [6]. In addition to the low TMB, various other immunosuppressive mechanisms, such as wingless and int-1 (WNT) and Kirsten rat sarcoma viral oncogene homolog (KRAS) signaling, low neoantigen expression, and human leukocyte antigen (HLA)-I loss, can contribute to immunotherapy resistance [7–11].

Combination ICB treatment with anti-cytotoxic T-lymphocyte associated protein 4 (CTLA-4) and anti-PD-1 exhibited enhanced anti-tumor activity in some tumors, including melanoma [12], hepatocellular carcinoma [13], and MSI-H CRC [14]. Again, MSS CRC showed poor responses to combination treatments using conventional anti-CTLA-4 antibodies, ipilimumab [15,16], and tremelimumab [17]. Interestingly, encouraging objective responses in MSS CRC have been observed in recent early-phase clinical trials of the dual targeting approach using the novel anti-CTLA-4 antibodies, botensilimab [18], and muzastotug [19]. While CTLA-4 blockade is actively investigated in clinical trials of MSS CRC, a deeper understanding of how CTLA-4 is involved in the immune evasion of MSS CRC is still required.

Tumor protein 53 (*TP53*) is the most commonly mutated tumor suppressor gene in cancer and one of the most frequently mutated genes in MSS CRC [20]. *TP53* mutation is important for the malignant transformation of tumor cells, but can also modulate the tumor microenvironment (TME) to promote immune evasion and metastasis [8,21–23]. However, the immunomodulatory mechanism of *TP53* mutations in CRC remains poorly understood.

*CEBPB* encodes CCAAT/enhancer binding protein β (C/EBPβ), which is a transcription factor with a basic leucine zipper domain (bZIP) involved in inflammatory and immune responses [24,25]. Previous studies have demonstrated its role as an immunomodulator in TME. *CEBPB* expression in myeloid-derived suppressor cells (MDSCs) was an important regulator for suppressing cytotoxic T cell functions and creating an immunosuppressive TME [26]. Moreover, tumor cell *CEBPB* expression was involved in modulating TME through MDSC and regulatory T cells (Treg) in breast cancer [27] and CRC [28] models, respectively.

Here, we performed single-cell RNA sequencing (scRNA-seq) of CRC patient tumor tissues and transformation-related protein 53 (*Trp53*) knockout (KO) syngeneic mouse tumors to investigate the influence of tumor cell mutations and gene expression on immune cell gene expression and TME composition in CRC.

## Materials and Methods

### Cell lines

Human CRC cell lines [HCT116 (*TP53* WT), Caco-2 (E204*), SNUC1 (S166*), SNU81 (K132T and R213*), SNU1197 (R175H), SNU1411 (S94*), SNU1544 (WT), and SW480 (R273H and P309S)], human embryonic kidney cells (HEK293T), human T lymphoblasts (Jurkat), and mouse T lymphoblasts (EL4) were purchased from the Korean Cell Line Bank. The murine colon carcinoma cell lines [CT26.WT (*Trp53* WT) and MC38 (G242V and S258I)] and human colon carcinoma cell lines [SW837 (*TP53* R248W) and SW1463 (R248Q)] were purchased from the American Type Culture Collection. The CRC cell lines, Jurkat, and EL4 were cultured in Roswell Park Memorial Institute (RPMI)-1640 (LM011-01, Welgene), and HEK293T and MC38 cells were cultured in Dulbecco’s Modified Eagle Medium (LM001-05 for HEK293T and LM001-08 for MC38, Welgene) supplemented with 10% fetal bovine serum (FBS; S001-01, Welgene) and 1% penicillin–streptomycin (LS202-02, Welgene). Medium for MC38 was additionally supplemented with 10.1 mmol/l non-essential amino acids (LS-005-01, Welgene), 2 mmol/l glutamine (LS002-01, Welgene), and 10 mmol/l 4-(2-hydroxyethyl)-1-piperazineethanesulfonic acid (BB001-01, Welgene). All cells were maintained in a humidified atmosphere of 37 °C with 5% CO_2_, and mycoplasma contamination was routinely tested using the e-Myco Mycoplasma PCR Detection kit ver. 2.0 (25235, iNtRON Biotechnology) to confirm that all cells were free of mycoplasma contamination.

### Human and mouse tumor preparation

Analysis of human tissue was approved by the Institutional Review Board (IRB) of Seoul National University Hospital (IRB number: 2008-131-1150). Primary tumor tissues from CRC patients were collected during surgery between October 2020 and November 2022. Patients who had received any prior anti-cancer treatment were excluded. The 8th edition of the American Joint Committee on Cancer staging system was used. All patients provided written informed consent before surgery, and the Declaration of Helsinki in biomedical research involving human subjects was followed.

Murine colon carcinoma CT26.WT (hereafter CT26) cells were transiently transfected with pSpCas9(BB)-2A-GFP (PX458) (48138, Addgene) containing sgRNA targeting exon 4 (5′-AGTGAAGCCCTCCGAGTGTC-3′) of *Trp53*. Green fluorescent protein (GFP)-positive cells were sorted using FACSAria II (BD Biosciences) 48 h post-transfection, seeded into a 96-well plate (0.5 cells per well), and then expanded as single clonal cell lines. *Trp53* KO of the monoclonal cells was assessed using Western blotting and Sanger sequencing of exon 4 of *Trp53* from the genomic DNA. Whole-exome sequencing (WES) was performed to confirm the complete KO of the *Trp53*.

The mouse experimental protocol was approved by the Institutional Animal Care and Use Committee (IACUC) of Seoul National University (IACUC number: SNU-220502-02) and was performed in accordance with the guidelines of the IACUC. Female BALB/c mice (5 weeks old) were obtained from KOATECH and housed in temperature- and humidity-controlled facilities under a 12-h light/12-h dark cycle. The mice were provided with standard chow and water ad libitum. After a 1-week acclimation period, *Trp53* KO and control CT26 cells (5 × 10^5^ cells in 200 μl phosphate-buffered saline [PBS]) were subcutaneously injected into the right flank of each mouse. The mice were euthanized 14 days post-inoculation using CO_2_ in accordance with institutional animal care protocols. Tumors were surgically excised, dissociated, and processed for scRNA-seq as described below. Tumors from 2 independent experiments, each using different *Trp53* KO clones and CT26 controls, were sequenced separately and subsequently pooled for further bioinformatic analysis.

### Single-cell RNA sequencing

Fresh tumor tissues from CRC patients obtained during surgery or mouse tumors were cut into small pieces in RPMI-1640 medium, enzymatically digested using a Tumor Dissociation Kit (130-096-730, Miltenyi Biotec), and mechanically dissociated using gentleMACS Dissociators (Miltenyi Biotec) according to the manufacturer’s instructions. Single cells were harvested after passage through a 70-μm strainer and incubated in red blood cell lysis buffer (64010-00-100, BioGerms) for 10 min. After washing with PBS, cells were resuspended in RPMI-1640 medium for scRNA-seq.

Single cells were resuspended in PBS containing 0.04% bovine serum albumin (BSA) (BSAS 1.0, Bovogen Biologicals) at a concentration of 1 × 10^6^ cells/ml, and cell viability was assessed using acridine orange/propidium iodide dye. Single-cell libraries were prepared using the Chromium Next GEM Single Cell 5′ Reagent Kit v2 (PN-1000263, 10x Genomics). Briefly, single-cell suspension, 5′ gel beads, master mix, and partitioning oil were loaded onto the Chromium Next GEM Chip K and run on Chromium iX (10x Genomics) to generate 10,000 single-cell gel beads in emulsion (GEM) per sample, and allow GEM to undergo reverse transcription with poly (dT) primer. The resulting 10× barcoded, full-length cDNA was then purified from the reaction mixture and amplified via polymerase chain reaction (PCR) with primers against common 5′ and 3′ ends added during GEM reaction. For V(D)J amplification from cDNA, additional PCR amplification was performed using primers specific for the T cell receptor (TCR) constant region and subjected to enzymatic fragmentation and size selection to generate viable length fragments. The final 5′ gene expression library and V(D)J library were quantified via the KAPA Library Quantification kit (07960140001, Roche) according to the manufacturer’s protocol and then sequenced as paired-end 150 base pair reads on Novaseq 6000 (Illumina).

### scRNA-seq data analysis

The raw sequencing read data obtained from the single-cell suspension of 30 CRC patients and mouse tumors were demultiplexed and mapped to the GRCh38 human reference and mm10 mouse reference genomes using 10x Genomics CellRanger v3.1.0 (https://www.10xgenomics.com/support/software/cell-ranger/) [29], resulting in an expression matrix. The single-cell expression matrix was analyzed using a conventional pipeline implemented in the Seurat R package [30]. Low-quality cells were filtered out using 3 criteria (number of expressed genes > 200, number of expressed genes < 6,000, and mitochondrial RNA ratio < 25%). To identify cell types, we first integrated datasets from 30 patients using an anchor-based method. Subsequently, cell cycle phase scores were regressed. Additional integration using Harmony was applied to project cells into a shared embedding of cell types [31]. Unsupervised clustering was achieved by applying the Louvain algorithm on shared nearest-neighbor modularity optimization.

The Wilcoxon rank-sum tests were performed for each cluster to identify cell-type marker genes. The main cell types were annotated using cell-type markers, including epithelial cells, CD4^+^ T cells, CD8^+^ T cells, natural killer (NK) cells, B cells, plasma cells, fibroblasts, endothelial cells, and mast cells. Additionally, 5 cell subtypes were identified within each of the CD4^+^ and CD8^+^ T cell populations based on specific markers. We used the scRepertoire R package for assigning TCR clonotypes to each T cell [32]. T cells with a TCR sequence shared in 2 or more cells were defined as expanded T cells. Clonal size was defined as the number of cells having identical TCR sequences and classified as “Hyper” in the top 5% with clone size greater than 50, “High” between the top 25% and the top 5% with clone size 6 to 50, and “Low” in non-single cases with clone size 2 to 5. For each patient, we then calculated the proportion of expanded T cell clones.

Major cell types in the mouse dataset, including malignant cells, monocytes, neutrophils, dendritic cells (DCs), B cells, T cells, natural killer T (NKT) cells, stromal cells, and unknown cell types, were annotated using specific cell-type markers, with specific malignant clusters denoted as “Malignant 1” and “Malignant 2” using differential expression of cell cycle-associated genes. Additionally, the cell subtypes within the T/NKT cell population were identified based on distinct markers.

### Differentially expressed gene and correlation analysis

We used the Wilcoxon rank-sum tests to identify the differentially expressed gene (DEG) of each cell type between the following patient groups. Patients were grouped based on key molecular factors, including MSI status and adenomatosis polyposis coli (*APC*), *TP53*, and RAS mutation status, as well as the expression levels of specific marker genes, such as *CEBPB* in epithelial cells and *CTLA4* in T cells. *CEBPB*-high epithelial cells and *CTLA4*-high T cells were grouped by fitting a 2-component Gaussian mixture model (GMM) to each marker’s expression distribution and using the first intersection of the 2 curves for values > 0 as the cutoff [33]. Epithelial cells with a mean *CEBPB* expression > 1.23 were thus classified as *CEBPB*-high and those below this threshold as *CEBPB*-low, while T cells with a mean *CTLA4* expression > 1.73 were designated *CTLA4*-high and the remainder *CTLA4*-low. In parallel, each patient was assigned to a “high” or “low” group based on whether the average expression of the relevant marker within that cell type in the patient exceeded or fell below the corresponding cutoff value. *P* values were adjusted using Bonferroni correction for all DEG analyses. The difference in the average expression between the 2 groups was represented as the Log2 fold change (Log2FC). For the patient dataset, DEGs were identified using a Log2FC cutoff of 0.5 and an adjusted *P* value below 0.05.

To identify genes commonly up-regulated in *Trp53* KO compared to *Trp53* WT mouse cells, we performed DEG analysis between the 2 heterogeneous malignant groups. Among the genes with a Log2FC greater than 1.5, an adjusted *P* value less than 0.05, and expression detected in more than 25% of cells in each group, protein-coding genes that also exhibited significant differences in expression according to *TP53* mutation status in human tumors were identified.

To identify genes whose expression patterns in various cell types align with *CEBPB* expression in epithelial cells across patients, we computed Pearson’s correlation between the patient-level average expression of each gene in each cell type and the patient-level average expression of *CEBPB* in epithelial cells. We collected significantly correlated genes with an absolute Pearson’s correlation coefficient (*r*) greater than 0.25 and a *P* value less than 0.05. *P* values were adjusted by 100,000 permutations. To strengthen the statistical inference, we fitted linear mixed-effects models that accounted for patient-level clustering. For patient *i* and cell *j*, the model was *CTLA4_ij_* = *β*_0_ *+ β*_1_*CEBPB_i_*^(z)^ + *u_i_ + ε_ij_*, where *CTLA4_ij_* denotes the normalized *CTLA4* expression of cell *j* from patient *i*, *CEBPB_i_*^(*z*)^ is the patient-level mean *CEBPB* expression in epithelial cells, *z*-scored across patients, *u_i_ ~ N*(0, σ*_u_*^2^) is a patient-specific random intercept, and *ε_ij_ ~ N*(0, σ*_u_*^2^) is the residual error. The models were fit using maximum likelihood, and 2-sided *P* values for fixed effects were obtained with Satterthwaite’s degrees-of-freedom approximation.

We performed over-representation enrichment analysis with Enrichr [34,35] and gene set enrichment analysis (GSEA) with the fgsea R package [35] on the DEGs and *CEBPB*-correlated genes, utilizing gene sets from the Kyoto Encyclopedia of Genes and Genomes, Gene Ontology Biological Process, MSigDB, and Reactome. To assess lymphocyte activation in the TME, significant terms with a *P* value less than 0.05 from the enrichment analysis were visualized as a heatmap.

We calculated *r* between each patient’s proportion of expanded clones and the mean *CEBPB* expression in their epithelial cells to evaluate the association between epithelial *CEBPB* expression and T cell clonal expansion.

### WES and MSI test

Formalin-fixed paraffin-embedded (FFPE) tissue samples were used for WES. Representative tumor and normal tissue areas were selected, macrodissected on glass slides, and used to extract genomic DNA using the FFPE gDNA Miniprep System (A2351, Promega) following the manufacturer’s recommended procedures. Sheared DNA was processed using the SureSelect XT Human All Exon V5 Kit (5190-6209, Agilent). Indexed pooled libraries were sequenced on an Illumina NovaSeq 6000 system (Coverage—Normal: 200×, Tumor: 400×). Sequencing reads were mapped to the human reference genome using both hg19 and GRCh38. Somatic variants were called using the GATK-Mutect2 tool from matched normal and tumor pairs. Several genetic characteristics, including somatic mutations and TMB, were identified using downstream analysis tools. TMB was calculated by dividing the number of non-synonymous mutated bases by the megabase (Mb) of the tumor genome obtained from WES. TMB was categorized into TMB-high and -low groups using a cutoff of 10/Mb.

The MSI test was performed by evaluating 5 microsatellite markers (D2S123, D5S346, D17S250, BAT25, and BAT26). PCR products of each marker were electrophoresed and analyzed. MSI status was classified as MSI-high (MSI-H, instability at 2 or more markers), MSI-low (MSI-L, instability at a single marker), or microsatellite stable (MSS, no instability). MSI-L was grouped with MSS.

### Immunohistochemistry

Tissue sections were cut to a 4-μm thickness and placed on slides. Sections were stained using a BenchMark ULTRA automated immunohistochemistry (IHC) stainer (Roche Diagnostics). Detection was performed using the Ventana ChromoMap Kit (Roche Diagnostics). Sections were deparaffinized using EZ Prep solution (950-102, Roche Diagnostics). The CC1 buffer (950-124, Roche Diagnostics) containing Tris/borate/ethylenediaminetetraacetic acid (EDTA) (pH 8.4) was used for antigen retrieval. Endogenous peroxidase activity was blocked using 3% H_2_O_2_ for 4 min at 37 °C.

For human tissue staining of C/EBPβ and CTLA-4, primary antibodies (Table S1) were incubated for 32 min at 37 °C, and a secondary antibody for 20 min at 37 °C. Tissues were incubated in diaminobenzidine (DAB) and H_2_O_2_ substrate for 8 min at 37 °C followed by hematoxylin and bluing reagent (05266769001, Roche Diagnostics) counterstain at 37 °C. Tris buffer (pH 7.6) was used as the washing solution.

IHC quantification was performed using QuPath software (version 0.5; https://qupath.github.io/) with the Positive Cell Detection tool, which automatically identifies positive cells and measures the DAB intensity. Tumor epithelial and tumor-infiltrating lymphocyte (TIL) regions of interest were annotated by a pathologist. Staining intensity was categorized into 3 levels (1+, weak; 2+, moderate; and 3+, strong), and the histoscore (H-score) was calculated as H-score = (3 × % of 3+ staining cells) + (2 × % of 2+ staining cells) + (1 × % of 1+ staining cells), ranging in the final H-score from 0 to 300. C/EBPβ cytoplasmic and nuclear staining within epithelial cells was scored using this H-score. For CTLA-4, the counting score was defined as the mean number of CTLA-4-positive (staining intensity ≥1+) TILs per mm^2^. The H-scores and counting scores from multiple regions were averaged for each case [36,37].

### Public clinical dataset analysis

For The Cancer Genome Atlas (TCGA) CRC (colorectal adenocarcinoma Pan-Cancer Atlas: TCGA-COAD and TCGA-READ) dataset, clinical information, mRNA expression, and *TP53* mutation data were downloaded from cBioPortal (https://www.cbioportal.org/) [38,39]. mRNA expression was presented as Log2 (RNA-seq by expectation-maximization + 1), and *TP53* mutation status was curated using filters with somatic and pathogenic mutations, which were provided by cBioPortal. *TP53* mutations were further categorized into truncating (including nonsense and frameshift insertions or deletions) and missense mutations. Data on the consensus molecular subtype (CMS) of each sample were obtained from Synapse (https://www.synapse.org/, Sage Bionetworks) [40]. TCGA data for other tumors were also downloaded from cBioPortal. These include breast carcinoma (TCGA-BRCA), uterine corpus endometrial carcinoma (TCGA-UCEC), brain low-grade glioma (TCGA-LGG), pancreatic adenocarcinoma (TCGA-PAAD), head and neck squamous cell carcinoma (TCGA-HNSC), and stomach adenocarcinoma (TCGA-STAD).

For the Sidra-Leiden University Medical Center Atlas and Compass of Immune Cancer Microbiome Interactions (LUMC AC-ICAM) CRC dataset (briefly, Sidra-LUMC) [41], clinical information, mRNA expression, CMS class, and *TP53* mutation data were downloaded from cBioPortal. Patients with stage IV disease were excluded from the survival analysis, considering the heterogeneity of clinical features, such as performance status, disease burden, and metastatic sites, and treatments, including chemotherapy, that could affect survival. *CEBPB*-high and -low samples were divided based on the median *CEBPB* expression. In the quartile group analysis, patients were grouped into 4 groups based on quartiles, where Q1 represents the lowest 25% of *CEBPB* expression and Q4 the highest 25%.

To assess spatial TIL densities of whole-slide images (WSIs) from the TCGA-CRC dataset, an artificial intelligence-powered model, Lunit SCOPE IO (Lunit), was used for classifying immune phenotypes (IPs) of the TME [42]. Spatial TIL densities were analyzed by detecting cells and segmenting tissue regions in hematoxylin and eosin (H&E)-stained WSI. Each WSI, varying in size, was divided into grids of 0.25 mm^2^ for a detailed analysis. The model estimated TIL densities and classified the IP of each grid based on the following criteria: inflamed IP, defined as intratumoral TIL density ≥ 130/mm^2^; immune-excluded IP, defined as intratumoral TIL density < 130/mm^2^ and stromal TIL density ≥ 260/mm^2^; and immune-desert IP, defined as TIL densities below the threshold in both areas. The overall WSI-level Inflamed Score was defined as the proportion of grids in each IP class among the total grids analyzed in the WSI. The H&E-stained WSI of colon cancer (COAD) and rectal cancer (READ) included in the TCGA-CRC dataset were downloaded from The Cancer Imaging Archive of the National Institutes of Health of USA (https://www.cancerimagingarchive.net/) [43].

### Public scRNA-seq and spatial transcriptomics data analysis

We re-analyzed a publicly available scRNA-seq dataset of 62 CRC patients from Gene Expression Omnibus (GEO) (https://www.ncbi.nlm.nih.gov/geo/query/acc.cgi?acc=GSE178341) and calculated the correlation between *CEBPB* expression in epithelial cells and *CTLA4* expression in other cell types. To derive cell type-specific expression profiles, we used the predefined annotations provided in the original study [44]. For CD8^+^ exhausted T cell (Tex) annotation, we re-analyzed pre-annotated CD8^+^ T cells using the Louvain clustering algorithm implemented in the Seurat R package [30]. We annotated cluster 3 as CD8^+^ Tex cells based on the expression of *CTLA4*, hepatitis A virus cellular receptor 2 (*HAVCR2*), lymphocyte-activating 3 (*LAG3*), programmed cell death protein 1 (*PDCD1*), and related genes.

We downloaded a publicly available Visium dataset of CRC patients comprising 14 slides [45] and calculated the correlation between *CEBPB* and *CTLA4* expression in 4 selected slides (SN124_A938797_Rep2, SN048_A121573_Rep2, SN123_A551763_Rep1, and SN048_A416371_Rep2) with *CTLA4*-expressing spots exceeding 5%. To extend the range of correlation calculation, we generated pseudo-spot data by summing the expression values of each spot and its 6 surrounding neighbors, reflecting the hexagonal layout of the Visium platform. Pseudo-spots comprising fewer than 4 spots were excluded to minimize bias. Each Visium slide’s spots were classified into 4 distinct groups based on the expression levels of *CEBPB* and *CTLA4*. Classification was performed using the median expression value of each gene as a threshold.

### RNA sequencing

Total RNA from CT26 cells stably overexpressing *Cebpb*, as well as from SNU81 and SNU1544 cells transiently overexpressing *CEBPB*, was extracted using the RNeasy Kit (74104, Qiagen) or TRI Reagent (TR118, Molecular Research Center) according to the manufacturer’s instructions. RNA concentration was quantified using a NanoDrop instrument (Thermo Fisher Scientific), and RNA integrity was assessed using a 2100 Bioanalyzer (Agilent). RNA sequencing libraries were constructed using TruSeq Standard mRNA library preparation kit (Illumina), and sequencing was performed in 150-bp pair-end mode on NovaSeq6000 systems (Illumina). The RNA reads were aligned to the human (GRCh38) or mouse (mm10) reference genome and quantified as transcripts per million (TPM).

Public RNA expression data of the CRC cell lines (Expression Public 24Q2) were obtained from the DepMap portal (https://depmap.org/portal/). In total, 80 CRC cell lines were selected, and the expression levels of *CEBPB* and lipocalin-2 (*LCN2*) were analyzed to assess their correlation using GraphPad Prism 8 (GraphPad Software).

### Manipulation of gene expression

To establish stable knockdown cell lines*,* sense and antisense gene-specific short hairpin RNA (shRNA) oligos for *Trp53* and *Cebpb* (Table S1) were annealed and ligated into the pLKO.1 lentiviral vector (10878, Addgene). The lentivirus was generated in HEK293T cells by co-transfection with pMD2.G (12259, Addgene) and psPAX2 (12260, Addgene). Puromycin selection was performed for 2 weeks after lentiviral infection, and polyclonal cells were used for subsequent experiments. To silence *TP53*, gene-specific and control small interfering RNA were purchased from Bioneer and transfected into CRC cancer cell lines using Lipofectamine RNAiMax (13778075, Invitrogen) according to the manufacturer’s instructions.

For overexpression of genes, cDNA of human *TP53* and *LCN2* from HEK293T and mouse *Lcn2* from CT26 were generated by PCR amplification, cDNA was obtained from human *CEBPB* (SC319561, OriGene) and mouse *Cebpb* (MR227563, OriGene), open reading frame clones were cloned into entry vector pDONR221 (12536017, Thermo Fisher Scientific), and entry vector with non-transcribed stuffer sequence (C125-E01, a gift from Dominic Esposito, Frederick National Laboratory for Cancer Research) was used for empty vector (EV) controls. The oligonucleotide sequences used for cloning are listed in Table S1. *TP53* R175H mutant vector was generated by PCR using Pfu High-Fidelity DNA Polymerase (600380, Agilent) with mutant-specific primer pairs described in Table S1 according to the manufacturer’s instructions. For transient overexpression, entry vectors harboring the gene of interest and the cytomegalovirus (CMV) promoter (C413-E36, provided by Dominic Esposito) were subcloned into the destination vector pDEST302 using a Gateway cloning kit (11789020, Thermo Fisher Scientific). The transfection was performed using Lipofectamine 3000 (L3000015, Invitrogen). The pLEX-FLAG destination vector (a gift from Lucy Young, University of California, San Francisco) was used for the stable lentiviral expression system. The lentiviral destination vector pCW57.1 (41393, Addgene) was used for doxycycline-inducible overexpression in the cell lines. After puromycin selection for 2 weeks, the culture medium was replaced with RPMI-1640 supplemented with 10% Tet system-approved FBS (631107, Takara Bio). Cells were then treated with 1 μg/ml doxycycline for 48 h and then examined in subsequent analyses. A Sleeping Beauty transposon (SB)-based expression system was also used to generate stable expression cell lines. Gateway cloning was performed using pSBDEST.H (79464, Addgene) and the CMV promoter vector. After co-transfection with pCMV(CAT)T7-SB100 (34879, Addgene), selection was performed using hygromycin for 2 weeks. All constructs were verified by Sanger sequencing.

### Real-time PCR

Total RNA was isolated from cells using TRI Reagent. mRNA was reverse-transcribed using a first-strand cDNA synthesis kit (AE301-02, TransGen), and analyzed using real-time PCR with SYBR green-based qPCR master mix solution (Takara Bio) with gene-specific primers. All real-time PCR assays were performed on an Applied Biosystems StepOnePlus Real-Time PCR system (Thermo Fisher Scientific) using a thermocycler with the following conditions: preincubation at 95 °C for 30 s; 45 cycles of 95 °C for 5 s, and 62 °C for 1 min. Melting curve analysis was used to determine the reaction specificity. Relative mRNA expression data were quantified using the 2^(−ΔΔCt)^ method and normalized to glyceraldehyde 3-phosphate dehydrogenase (*GAPDH*) or actin beta (*ACTB*). The primers used are listed in Table S1.

### Western blotting

The cells were lysed in radioimmunoprecipitation assay buffer (89900, Thermo Fisher Scientific) supplemented with protease and phosphatase inhibitors (78441, Thermo Fisher Scientific). Protein lysates were resolved using a polyacrylamide NuPAGE 4% to 12% Bis-Tris gel (NP0336BOX, Thermo Fisher Scientific) and transferred to a nitrocellulose membrane using the iBlot Transfer Stack (IB23001, Thermo Fisher Scientific). Membranes were incubated with primary antibodies overnight at 4 °C, washed using Tris-buffered saline containing 0.01% Tween-20, and incubated with horseradish peroxidase-conjugated secondary antibodies for 1 h at room temperature. After washing, the membrane was exposed to Chemiluminescent Substrate (34577, Thermo Fisher Scientific), and protein signals were detected using the iBright1500 system (Invitrogen). Detailed information on the primary and secondary antibodies is listed in Table S1.

### Chemical activation of p53

CRC cell lines harboring *TP53* WT, nonsense, or missense mutations were seeded in 6-well plates at a density of 0.5 to 0.75 × 10^6^ cells per well. On the following day, cells were treated with 10 μmol/l nutlin-3a (S8059, Selleckchem) or vehicle control (dimethyl sulfoxide) for 24 h. Additionally, SNU1544 cells (0.75 × 10^6^/well) were seeded in 6-well plates, and the following day, nutlin-3a (10 μmol/l) was added for 16 h. To further activate transcription of *CEBPB*, cells were treated with the cyclic adenosine monophosphate (cAMP) analog 8-bromoadenosine 3′,5′-cyclic monophosphate sodium salt (8-Br-cAMP; B7880, Sigma-Aldrich) for an additional 8 h. After treatment, the cells were washed twice with cold PBS, and protein and mRNA levels were analyzed by Western blotting and quantitative real-time PCR, respectively.

### Protein stability assay

To investigate the effects of p53 on C/EBPβ protein stability, CT26 cells stably expressing shCtrl or sh*Trp53* (0.75 × 10^6^/well) were seeded in 6-well plates. The following day, cells were treated with 5 μg/ml of cycloheximide (C4859, Sigma-Aldrich) for 2, 4, or 6 h. SNU1544 cells (0.75 × 10^6^/well) were seeded in 6-well plates. The following day, cells were pretreated with 10 μmol/l nutlin-3a as a *TP53* inducer, followed by treatment with 2 μmol/l MG132 (474790, Sigma-Aldrich) for 3 h. After treatment, cells were washed with cold PBS and harvested for Western blotting.

### Proliferation and soft agar colony formation assay

CT26 cells (4 × 10^3^/well) were seeded in 96-well plates and cultured for 24, 48, and 72 h. Cell viability was assessed using CellTiter-Glo (G9682, Promega), and luminescence was measured using a GloMax Discover Microplate Reader (Promega) according to the manufacturer’s instructions. For the colony formation assay, CT26 cells (2 × 10^3^) were mixed with sterile 1% agar solution (final concentration, 0.4%) and plated on 1% base agar in 24-well plates. Cells embedded in the top agar were cultured for 3 weeks, and the medium was changed twice a week. After incubation, colonies were stained with 400 μg/ml 3-(4,5-dimethylthiazol-2-yl)-2,5-diphenyltetrazolium bromide (MTT; 475989, Sigma-Aldrich) solution at 37 °C for 1 h, and the number of colonies was quantified by ImageJ software (National Institutes of Health of USA).

### Syngeneic mouse tumor model

The following mouse experiments were approved by the Seoul National University IACUC (IACUC number: SNU-220502-09). For the syngeneic immunocompetent tumor model, female BALB/c and C57BL/6N mice (5 to 6 weeks old) were obtained from KOATECH. After a 1-week acclimation period, a total of 8 × 10^5^ CT26 cells expressing either *Cebpb*-overexpression (oe) vector or EV, suspended in 100 μl of PBS, were subcutaneously injected into the right flanks of BALB/c mice. Similarly, 2 × 10^6^ MC38 cells with shRNA-mediated knockdown of *Cebpb* (sh*Cebpb*) or a non-targeting control shRNA (shCtrl), were suspended in 100 μl of PBS and injected into the right flanks of C57BL/6N mice. Tumors and spleens were surgically removed at approximately 21 days post-inoculation for immune cell analysis.

For the immunodeficient tumor model, female BALB/c nude mice (6 weeks old) were obtained from KOATECH and acclimated for 1 week. A total of 8 × 10^5^ CT26 cells expressing either *Cebpb*-oe or EV (in 100 μl PBS) were subcutaneously injected into the right flanks of the mice.

For in vivo ICB treatment, *Cebpb*-oe CT26 cells were injected subcutaneously into the flanks of immunocompetent BALB/c mice, as described above. When tumor volumes reached 75 to 125 mm^3^, the mice were randomized into 4 treatment groups: (a) anti-mouse CTLA-4 (BP0032, clone UC10-4F10-11, Bio X Cell), (b) anti-mouse PD-1 (BE0146, clone RMP1-14, Bio X Cell), (c) a combination of anti-CTLA-4 and anti-PD-1, and (d) control antibodies. Rat IgG2a (BE0089, Bio X Cell) and Armenian hamster IgG (BE0091, Bio X Cell) were used as isotype control antibodies. All treatments were administered intraperitoneally at 10 mg/kg every 3 days for a total of 6 doses.

Tumor volumes were measured twice per week using calipers and were calculated using the following formula: tumor volume (mm^3^) = length × width^2^ × 0.5. The tumor growth rates were compared between the groups. The mice were euthanized when the tumor volume reached approximately 1,000 to 1,500 mm^3^.

### In vitro co-culture system

To prepare splenic T cells, the spleens of 7- to 10-week-old BALB/c mice were dissected, gently mashed on a 70-μm cell strainer, and collected in RPMI-1640 medium. The splenic cells were centrifuged at 300 ×*g* for 7 min at 4 °C and then incubated in red blood cell lysis buffer for 10 min at room temperature. Following washing with cold PBS, T cells were isolated using Pan T cell isolation II (130-096-535, Miltenyi Biotec) according to the manufacturer’s protocols. Before co-culturing with cancer cells, isolated T cells (1 × 10^6^) were pre-incubated with plate-coated anti-mouse CD3ε (1.25 μg/ml; BE0001-1, Bio X Cell) and soluble anti-mouse CD28 (1 μg/ml; BE0015-1, Bio X Cell) for polyclonal T cell activation, and seeded at a density of (2 × 10^5^) cells on 96-well plates. One hour later, the indicated CT26 cells were added to T cells at ratios of 5:1 or 10:1 (T cells:CT26 cells). In LCN2 neutralization experiments, cells were treated with 2 μg/ml lipocalin-2 neutralizing antibody (ab1857LC-050, R&D Systems) prior to co-culture with T cells. Co-culture was maintained for 48 or 72 h in RPMI-1640 growth medium supplemented with 10% FBS, 1% antibiotics, and 55 μmol/l 2-mercaptoethanol (63689, Sigma-Aldrich), and subsequently subjected to flow cytometry analysis as described below.

Additionally, T lymphoblasts were co-cultured with CRC cells using a Transwell system. Briefly, Jurkat cells (3.5 × 10^6^) were stimulated with plate-coated anti-human CD3ε (5 μg/ml) and soluble anti-human CD28 antibodies (3.5 μg/ml; CDE-M120a and CD8-M120b, ACROBiosystems) for polyclonal T cell activation. SNU1544 or SW837 cells (5 × 10^5^ cells/well) were pre-seeded into the outer wells of a 12-well plate. The next day, the activated Jurkat cells (5 × 10^5^) were plated into the inner wells of a 0.4-μm pore-size transwell insert (3401, Corning). For Jurkat and SW837 co-culture, the cells were treated with 1 μg/ml of lipocalin-2 neutralizing antibody (ab1757LC-050, R&D Systems) and cultured in RPMI-1640 growth medium for 24 h. For the co-culture of mouse T lymphoblast EL4, CT26 cells (2 × 10^5^/well) were pre-seeded into 6-well plates, and EL4 (2 × 10^5^) were added into the inner wells of a 0.4-μm pore-size transwell insert (Corning) at a 1:1 ratio after 18 h and were maintained in RPMI-1640 growth medium for 48 h. T lymphoblast samples were harvested for real-time PCR analysis as described above.

### T cell proliferation assay

For the T cell proliferation assay, 1 × 10^7^ splenic T cells, as isolated above, were resuspended in 500 μl of PBS containing 10 μmol/l carboxyfluorescein succinimidyl ester (CFSE) (423801, BioLegend) and incubated at room temperature for 10 min. The staining was quenched by adding culture medium, followed by centrifugation at 300 ×*g* for 7 min at 4 °C. The cells were then resuspended in RPMI-1640 medium and co-cultured with CT26 cells as described above. After 72 h of incubation, the cultured cells were dissociated with trypsin-EDTA, and the CFSE dilution was analyzed by flow cytometry.

### T cell migration assay

CT26 cells were seeded at 5 × 10^5^/well onto 6-well plates and maintained for 48 h. Conditioned media (CM) were collected, centrifuged at 400 ×*g* for 5 min at 4 °C, and filtered through a 0.2-μm filter. A total of 5 × 10^5^ EL4 or mouse splenic T cells were seeded into transwell inserts with 8-μm pore size or 3-μm pore size, respectively. The bottom of the plate was filled with 600 μl of the indicated CM or fresh growth medium as a control and supplemented with 200 ng/ml of recombinant mouse C-X-C motif chemokine ligand 12 (CXCL12) (250-20A, Thermo Fisher Scientific). After 24 h, the cells in the bottom well and upper insert were counted using an automatic cell counting Countess 3 system (Thermo Fisher Scientific), and the transwell membrane was stained with hematoxylin. The percentage of migrating cells (%) was calculated as the number of cells in the bottom well/(number of cells in the upper insert + bottom well) × 100%.

### Flow cytometry

Mouse spleen cell suspensions were prepared by gently mashing through a 70-μm cell strainer, collected in RPMI-1640 medium, centrifuged at 300 ×*g* for 7 min at 4 °C, and incubated in red blood cell lysis buffer for 10 min at room temperature. To prepare mouse tumor single-cell suspensions, tumor samples were enzymatically dissociated using a Tumor Dissociation kit and mechanically dissociated using gentleMACS Dissociators, according to the manufacturer’s protocol. After the removal of red blood cells, the cells were stained with Live/Dead staining dye (L34942, Thermo Fisher Scientific) on ice for 30 min. Then, tumor and splenic cells (1 × 10^6^) were suspended in 50 μl of FACS buffer (PBS supplemented with 0.5% BSA and 2 mmol/l EDTA) and incubated with mouse anti-CD16/32 (FcgRII/III block; BioLegend) on ice for 10 min. To prepare co-cultured mouse spleen T cells and CT26 cells, the cells were harvested using 0.05% Trypsin-EDTA and washed twice with cold PBS. After centrifugation at 300 ×*g* for 7 min at 4 °C, the cell pellet was resuspended in the FACS buffer. Cell surface staining was performed for 30 min at 4 °C with anti-mouse antibodies listed in Table S1. After antibody incubation, the cells were washed with PBS, fixed with 1.6% paraformaldehyde for 15 min at room temperature for fixation of cells, and permeabilized with 0.7% Tween-20 in PBS for 15 min at room temperature. Intracellular antigen staining was performed for 30 min at room temperature, followed by washing with the FACS buffer. The cells were detected using a BD FACSymphony A5 (BD Biosciences), and the data were analyzed using FlowJo software v10.8.1 (BD Biosciences). Flow cytometry gating strategies are presented in Fig. S1.

### Cytokine array

A total of 1 × 10^6^ cells were seeded in 100-mm culture dishes. The following day, the cells were washed with PBS and cultured for 24 h in a serum-free medium. CM was concentrated using an Amicon Ultra centrifugal filter 3K device (UFC500324, MilliporeSigma) and applied to a mouse cytokine array (ARY028, R&D Systems), according to the manufacturer’s protocol, to assess the levels of 111 cytokines and chemokines. The signals were visualized using a chemiluminescence substrate, and mean intensity was analyzed using iBright1500 and iBright Analysis Software (Invitrogen) and normalized against internal reference controls.

### Statistical analysis

In vitro and in vivo data were analyzed using GraphPad Prism version 8. Data are presented as means ± standard deviation (SD), and the Shapiro–Wilk test was performed to determine if the data followed a normal distribution. Statistical comparisons were made using 2-tailed unpaired Student’s *t* tests, and *P* values < 0.05 were considered statistically significant. Statistical analysis of the publicly available data was performed using R software (version 4.3.1). To evaluate the differences in *CEBPB* expression between the groups, the Wilcoxon rank-sum test was used. The correlation between 2 continuous variables was evaluated using Spearman’s correlation. Progression-free survival (PFS) and disease-specific survival (DSS) curves were estimated using the Kaplan–Meier method and compared statistically using the log-rank test with the ggsurvplot function of the R package survminer (version 0.4.9). The survival times, which are the number of months between the date of surgery and the date of the event or the last day of follow-up, were downloaded from cBioPortal. Multivariate Cox regression analysis was conducted to obtain hazard ratios (HRs) and corresponding *P* values by incorporating conventional clinical and biological variables (age, sex, stage, tumor location, and TMB) using the coxph function of the R package survival (version 3.7-0). The results were visualized with the forestplot package (version 3.1.3).

## Results

### CRC tumor microenvironment and mutations

**Fig. 1.**
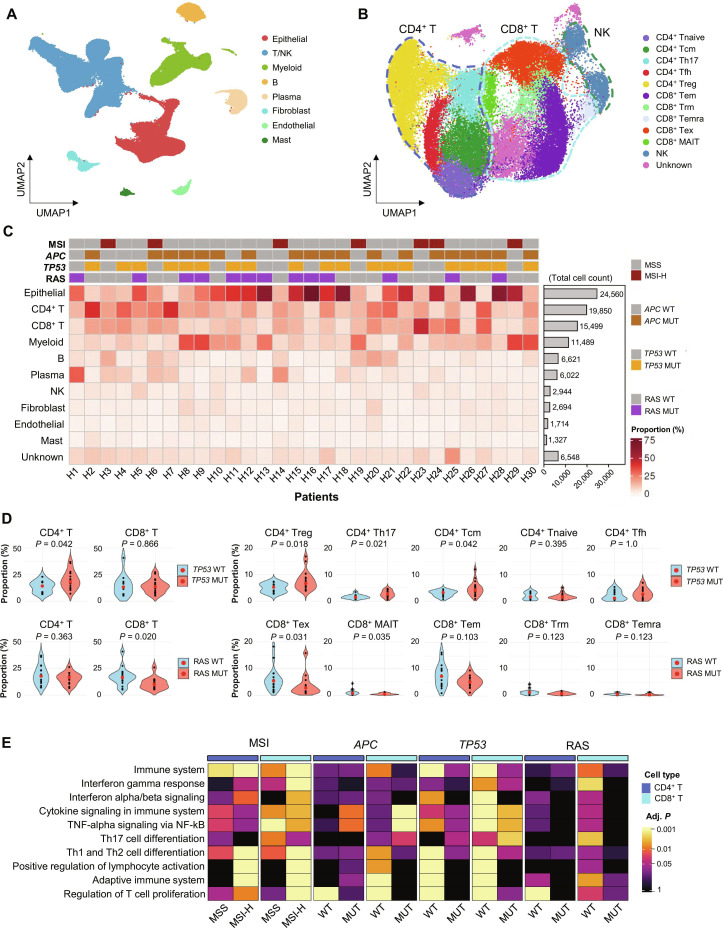
TME profile of tumor tissues from 30 CRC patients using scRNA-seq. (A) A total of 99,268 cells derived from scRNA-seq of tumor tissues from 30 CRC patients were clustered and annotated to identify main cell types, as displayed in UMAP. (B) Subclustering and annotation of T/NK cell subtypes. (C) Heatmap showing proportions of each cell type per patient, with MSI status, and *APC*, *TP53*, and RAS mutation status, and bar graph displaying the total cell counts of the main cell types. (D) Violin plots showing proportions of CD4^+^ and CD8^+^ T cells according to *TP53* and RAS mutation status (left), and proportions of CD4^+^ T cell subtypes according to *TP53* mutation and CD8^+^ T cell subtypes according to RAS mutation (right). *P* values were obtained using the Wilcoxon rank-sum test. Red dots indicate the median. (E) Heatmap showing representative examples from the pathway enrichment analysis of DEGs from T cells according to MSI and mutation statuses*.* Abbreviations: Adj. *P*, adjusted *P* value; *APC*, adenomatosis polyposis coli; CRC, colorectal cancer; DEG, differentially expressed gene; MAIT, mucosal-associated invariant T cell; MSI, microsatellite instability; MSI-H, microsatellite instability-high; MSS, microsatellite stable; MUT, mutation; NF-κB, nuclear factor kappa B; NK, natural killer cell; RAS, rat sarcoma virus; scRNA-seq, single-cell RNA sequencing; T/NK, T cell/natural killer cell; Tcm, central memory T cell; Tem, effector memory T cell; Temra, terminally differentiated effector memory T cell; Tex, exhausted T cell; Tfh, T follicular helper cell; Th1, T helper 1; Th2, T helper 2; Th17, T helper 17 cell; TME, tumor microenvironment; Tnaive, naive T cell; *TP53*, tumor protein 53; Treg, regulatory T cell; Trm, tissue-resident memory T cell; TNF-alpha, tumor necrosis factor-alpha; UMAP, uniform manifold approximation and projection; WT, wild type.

scRNA-seq and WES were performed with primary tumor tissue from 30 CRC patients (Tables S2 and S3). In scRNA-seq, a total of 99,268 cells were partitioned into 14 unsupervised clusters and annotated into 8 main cell types based on canonical markers (Fig. 1A and Fig. S2A). T/NK cells were further partitioned into 27 unsupervised clusters, which were then merged into 12 cell subtypes (Fig. 1B and Fig. S2B). The mutation profile was as expected for CRC, with *APC* (63.3%) and *TP53* (63.3%) being the most frequently mutated genes, and RAS (*KRAS* and *NRAS*) mutations in 43.3% of patients. The MSI status was high in 23.3% (*n* = 7) of patients, and all TMB-high tumors (*n* = 7) were MSI-H (Fig. 1C and Table S2).

We first analyzed the cellular composition according to the MSI status and common somatic mutations to examine the influence of the tumor cell mutation on the immune microenvironment (Fig. 1C and Fig. S3). A higher proportion of CD4^+^ T cells was observed in *TP53* mutant tumors, whereas CD8^+^ T cells were decreased in RAS mutant tumors (Fig. 1D). Among the CD4^+^ T cell subtypes, Treg, T helper 17 cells (Th17), and central memory T cells (Tcm) were higher in *TP53* mutant tumors than in *TP53* WT tumors; CD8^+^ Tex and mucosal-associated invariant T cells (MAIT) were lower in RAS mutant tumors than in RAS WT tumors (Fig. 1D).

Next, we analyzed DEGs of each cell type according to the genetic status of the tumor (Fig. S4A and Table S4). In the pathway analysis of DEGs, changes in immune-associated pathways were more frequently observed in the analyses according to MSI and *TP53* mutation status (Fig. S4B and Table S5). As expected, immune activation-related pathways, such as “immune system” and “interferon gamma response”, were enriched in the DEGs of T cells from MSI-high tumors compared to MSS (Fig. 1E). In contrast, they were depleted in the DEGs of T cells from *TP53* mutant tumors compared to *TP53* WT tumors (Fig. 1E), suggesting that *TP53* mutations might be associated with immune evasion by negatively affecting T cells.

### *TP53* mutation and *CEBPB* overexpression in tumor cells

**Fig. 2.**
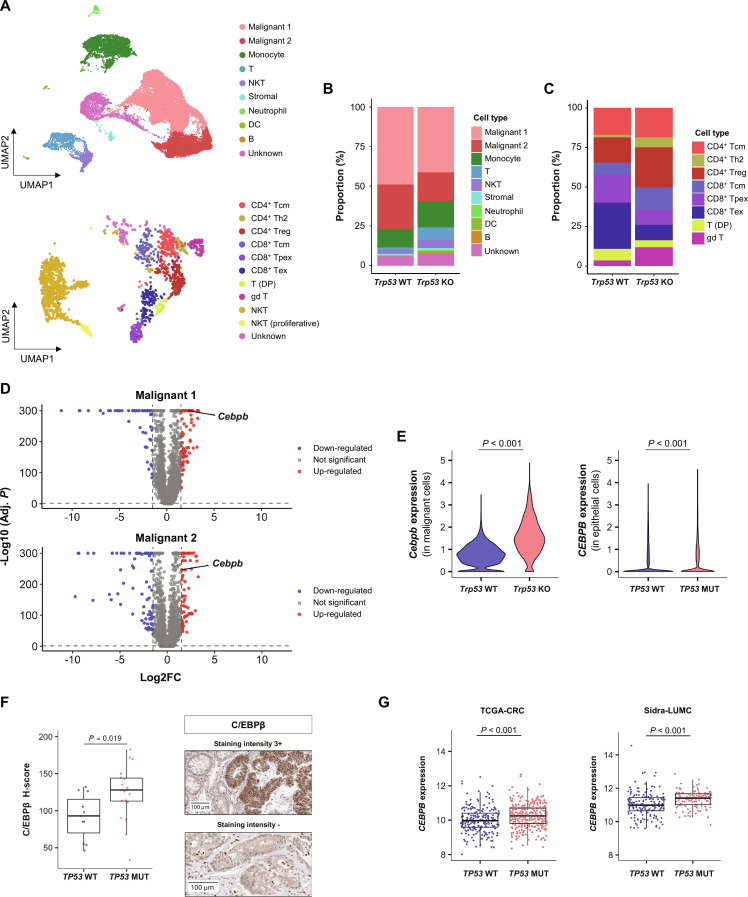
*TP53* mutation is associated with elevated *CEBPB* expression. (A) scRNA-seq was performed on mouse tumors grown after subcutaneous injection of control (*Trp53* WT) and *Trp5*3 KO CT26 cells into syngeneic BALB/c mice (*n* = 2). Clustering and annotation showing main cell types (top), and subclustering and annotation of T and NKT cell subtypes (bottom). (B and C) The proportion of main cell types (B) and T cell subtypes (C) in control (*Trp53* WT) and *Trp53* KO CT26 tumors. (D) Volcano plots showing DEGs identified from *Trp53* KO compared to control CT26 tumors in Malignant 1 and Malignant 2 cell types. (E) Violin plots showing *Cebpb* transcript level in mouse malignant single cells according to *Trp53* KO status (left), and *CEBPB* transcript level in human epithelial single cells according to *TP53* mutation status from scRNA-seq data of 30 CRCs (right). (F) IHC analysis of C/EBPβ was performed on CRC tumor tissues from 30 patients. Box plot comparing the H-score of C/EBPβ expression in epithelial cells between *TP53* WT (*n* = 11) and *TP53* MUT (*n* = 19) patient groups (left). Representative IHC images of epithelial tumor cells with strong (3+) C/EBPβ staining (top right) and negative (−) staining (bottom right). (G) Box plots showing *CEBPB* transcript levels according to *TP53* mutation status in the TCGA-CRC (left) and the Sidra-LUMC datasets (right). Box plots show upper and lower quartiles, median values as center lines, and whiskers extend to 1.5 × interquartile range. All *P* values were calculated using the Wilcoxon rank-sum test. Abbreviations: Adj. *P*, adjusted *P* value; *CEBPB*, CCAAT enhancer binding protein beta; DC, dendritic cell; DEG, differentially expressed gene; T (DP), double positive T cell; gd T, gamma delta T cell; H-score, histoscore; IHC, immunohistochemistry; MUT, mutation; NKT, natural killer T cell; Sidra-LUMC, Sidra-Leiden University Medical Center; Tcm, central memory T cell; TCGA-CRC, The Cancer Genome Atlas colorectal cancer; Tex, exhausted T cell; Th2, T helper 2; Tpex, progenitor exhausted T cell; *TP53*, tumor protein 53; *Trp53*, transformation related protein 53; *Trp53* KO, *Trp53* knockout; Treg, regulatory T cell; WT, wild type.

To investigate the impact of *TP53* mutation on the TME, we utilized the CT26 cell line and a BALB/c syngeneic mouse model. *Trp53* KO was performed using CRISPR/Cas9 in CT26 cells harboring WT *Trp53*. Tumors grown after subcutaneous implantation of control *Trp53* WT and *Trp53* KO CT26 cells in BALB/c mice were analyzed using scRNA-seq. By clustering the scRNA-seq data, we identified 12 unsupervised clusters (Fig. S5A) and then annotated them into 9 cell types, including 2 heterogeneous malignant cell types (Fig. 2A). Infiltration of immune cells, including monocytes, T cells, and NKT cells, was increased in *Trp53* KO tumors compared to that in control tumors (Fig. 2B). To profile lymphocyte compositional changes, we further resolved T and NKT cells (Fig. S5B). Notably, the increased infiltration of CD4^+^ Tregs aligned with the human scRNA-seq data of 30 CRCs (Figs. 2C and 1D).

We analyzed the DEGs of *Trp53* KO tumors in each cell type and identified 36 genes with elevated expression in both malignant cell populations (Fig. 2D and Table S6). Next, we evaluated the expression of the up-regulated genes according to *TP53* mutation status in the human scRNA-seq data. *Cebpb* was one of the genes with elevated expression in both mouse and human data (Fig. 2E and Fig. S6). The overexpression of *Cebpb* in *Trp53* KO CT26 cells was confirmed using Western blotting and real-time PCR (Fig. S7A). Restoration of WT p53 expression by transducing *Trp53* WT decreased C/EBPβ expression in *Trp53* KO cells (Fig. S7B), supporting that *Trp53* KO is the cause of the elevated *Cebpb* expression but not an off-target effect of CRISPR/Cas9.

We also confirmed the higher expression of C/EBPβ in *TP53* mutant tumors using IHC analysis of the 30 human CRC tumor tissues (Fig. 2F). Although *CEBPB* mRNA expression was also higher in tumor epithelial cells from RAS mutant tumors, no significant difference was observed in the IHC analysis (Fig. S7C and D).

Next, we expanded our analysis of *CEBPB* mRNA expression to publicly available TCGA-CRC and Sidra-LUMC datasets. *CEBPB* expression was significantly higher in CRC with *TP53* mutations in both the TCGA-CRC and Sidra-LUMC datasets (Fig. 2G), but not with RAS mutations (Fig. S7E). Elevated *CEBPB* expression was comparable between CRC patients with truncating or missense *TP53* mutants (Fig. S7F). Higher expression of *CEBPB* in *TP53* mutant tumors was also observed in several other cancers, including breast, endometrial, and pancreatic cancers, in the TCGA dataset (Fig. S7G).

### *CEBPB* up-regulation by *TP53* mutation

**Fig. 3.**
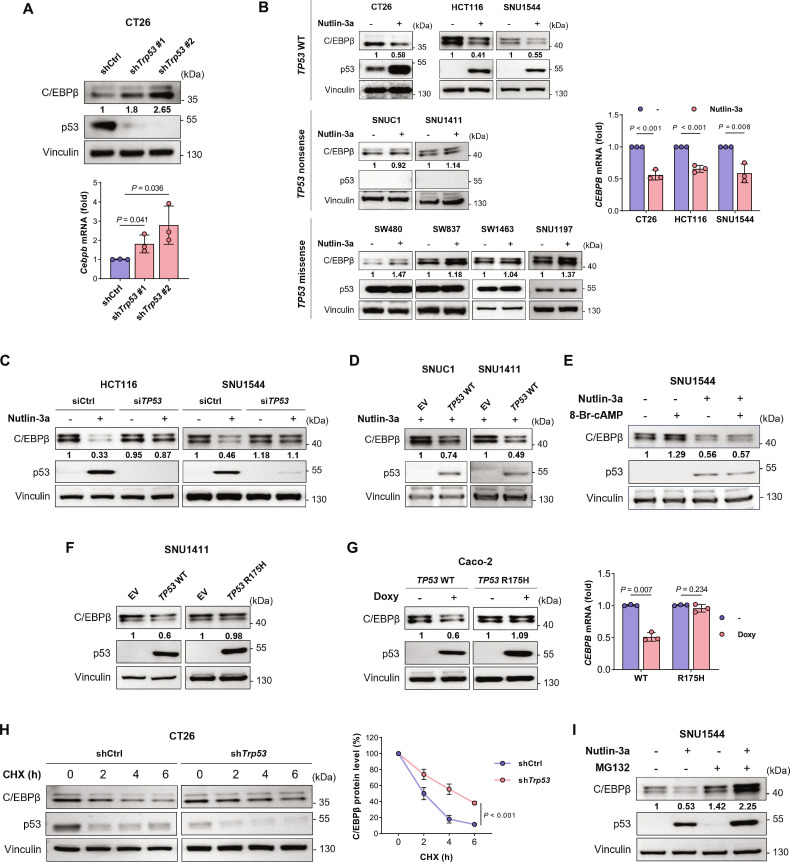
WT p53 suppresses mRNA and protein levels of C/EBPβ in CRC cell lines. (A) Protein and mRNA expression of C/EBPβ in CT26 cells stably expressing shCtrl or sh*Trp53* were analyzed by Western blotting and real-time PCR, respectively. (B) C/EBPβ protein expression was determined in CRC cell lines harboring *TP53* WT, nonsense mutation, and missense mutation treated with nutlin-3a (10 μmol/l) for WT p53 induction or vehicle for 24 h (left), as well as *CEBPB* mRNA levels in *TP53* WT cell lines (right). (C) C/EBPβ protein expression was compared in *TP53* WT HCT116 and SNU1544 cells after transfection with siCtrl or si*TP53*, followed by nutlin-3a (10 μmol/l) or vehicle treatment for 24 h. (D) C/EBPβ protein expression was determined in *TP53* nonsense SNUC1 and SNU1411 cells after transient transfection with EV or *TP53* WT, followed by 24 h of nutlin-3a (10 μmol/l) treatment. (E) C/EBPβ protein levels were determined in SNU1544 cells pretreated with nutlin-3a (10 μmol/l) for 16 h and stimulated with 8-Br-cAMP (100 μmol/l), an inducer of *CEBPB* transcription, for 8 h. (F) C/EBPβ protein levels were determined in SNU1411 cells transiently transfected with EV, *TP53* WT, or *TP53* R175H for 24 h. (G) C/EBPβ protein (left) and mRNA (right) levels were assessed in p53-null Caco-2 cells stably transduced with doxycycline-inducible *TP53* WT or *TP53* R175H following treatment with 1 μg/ml doxycycline for 48 h. (H) C/EBPβ protein stability was compared in shCtrl and sh*Trp53* CT26 cells after CHX treatment (5 μg/ml) for the indicated times. (I) SNU1544 were treated with nutlin-3a (10 μmol/l) for 21 h, followed by MG132 (2 μmol/l) for 3 h to block proteasomal degradation. Protein expression was analyzed by Western blotting, representative of 3 independent experiments, and the values presented underneath or in the graph (H) were generated by densitometric analyses using ImageJ. mRNA levels were determined using real-time PCR. The bar graphs represent the mean ± SD; each dot represents an independent biological replicate. *P* values were calculated using 2-tailed Student’s *t* tests. Abbreviations: 8-Br-cAMP, 8-bromoadenosine 3′,5′-cyclic monophosphate; *CEBPB*, CCAAT enhancer binding protein beta; CHX, cycloheximide; CRC, colorectal cancer; doxy, doxycycline; EV, empty vector; SD, standard deviation; shCtrl, control short hairpin RNA; sh*Trp53*, short hairpin RNA for *Trp53*; si*TP53*, small interfering RNA for *TP53*; WT, wild type.

The relationship between *TP53* and *CEBPB* was further validated by the down-regulation of p53 using shRNA, resulting in the elevation of C/EBPβ protein and *Cebpb* mRNA expression in CT26 (Fig. 3A). In contrast, up-regulation of p53 expression using nutlin-3a, a mouse double minute 2 homolog (MDM2) inhibitor, decreased *Cebpb* mRNA and C/EBPβ protein expression in CT26, as well as *TP53* WT human CRC cell lines HCT116 and SNU1544 **(**Fig. 3B). The down-regulation of C/EBPβ by nutlin-3a treatment was abrogated by si*TP53* (Fig. 3C), supporting that p53 overexpression, but not an off-target effect of nutlin-3a, is involved in the C/EBPβ down-regulation. In contrast, nutlin-3a treatment did not decrease C/EBPβ expression in CRC cell lines with missense or nonsense *TP53* mutations (Fig. 3B). Transient expression of *TP53* WT*,* in addition to the nutlin-3a treatment, decreased C/EBPβ expression in the *TP53* nonsense mutant cell lines (Fig. 3D). The negative regulation of C/EBPβ expression by WT p53 in CRC was also demonstrated using cAMP analog, a known inducer of C/EBPβ [46,47]. The induction of C/EBPβ by 8-Br-cAMP was suppressed with nutlin-3a, which increased WT p53 expression (Fig. 3E).

To examine the difference in *CEBPB* regulation between WT and missense mutant p53, *TP53* WT or *TP53* R175H was expressed in p53-null cell lines. Transient expression of *TP53* WT resulted in C/EBPβ down-regulation, whereas *TP53* R175H did not decrease C/EBPβ expression (Fig. 3F). This was confirmed using a doxycycline-inducible system in a p53-null Caco-2 cell line. The expression of *TP53* WT decreased C/EBPβ expression in both protein and mRNA levels, whereas *TP53* R175H had no effect (Fig. 3G). These results were supported by the high *CEBPB* expression levels among the *TP53* missense mutations compared to the WT in the public datasets (Fig. S7F). Therefore, the *TP53* missense mutation represents a loss-of-function mutation with respect to *CEBPB* regulation [48,49], as it is impaired in its ability to down-regulate *CEBPB* expression.

Next, we investigated whether C/EBPβ expression is also regulated at the protein stability level by p53 in addition to the transcriptional repression [49]. C/EBPβ protein stability was analyzed by conducting a pulse-chase experiment. The rate of C/EBPβ protein degradation following cycloheximide treatment decreased when p53 expression was suppressed using shRNA (Fig. 3H). In addition, proteasomal inhibition using MG132 increased the C/EBPβ protein level and blocked the C/EBPβ down-regulation by p53 induction after nutlin-3a treatment (Fig. 3I). These findings suggested that proteasomal degradation may be an additional mechanism of C/EBPβ regulation by WT p53.

### *CEBPB* expression and clinicopathological characteristics

**Fig. 4.**
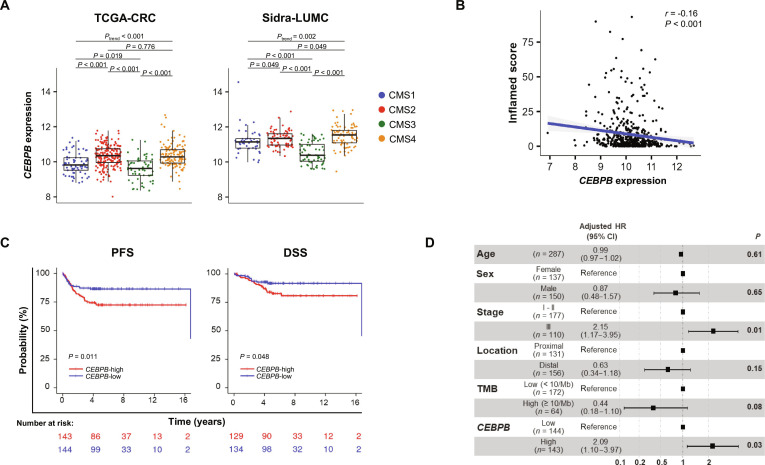
*CEBPB* expression and clinicopathological data. (A) Box plots of *CEBPB* mRNA expression according to CMS subtypes from the TCGA-CRC and Sidra-LUMC datasets. Box plots show upper and lower quartiles, median values as center lines, and whiskers extend to 1.5 × interquartile range. *P* values were calculated using the Wilcoxon rank-sum test. (B) Scatter plot comparing *CEBPB* expression and Inflamed Score measuring T cell infiltration using Lunit SCOPE IO from the TCGA-CRC dataset. The correlation coefficient (*r*) and *P* value were calculated using Spearman’s correlation analysis. (C) The Kaplan–Meier curves of PFS and DSS according to *CEBPB* expression in the Sidra-LUMC dataset. Stage I to III patients were included, and the median was used as the cutoff for *CEBPB* expression. *P* values were calculated using log-rank tests. (D) Forest plot of multivariate Cox proportional hazards analysis for PFS, presenting the adjusted HR and 95% CI. Abbreviations: *CEBPB*, CCAAT enhancer binding protein beta; CI, confidence interval; CMS, consensus molecular subtype; DSS, disease-specific survival; HR, hazard ratio; Mb, megabase; PFS, progression-free survival; Sidra-LUMC, Sidra-Leiden University Medical Center; TCGA-CRC, The Cancer Genome Atlas colorectal cancer; TMB, tumor mutational burden.

We analyzed *CEBPB* expression according to clinicopathological characteristics in the TCGA-CRC and Sidra-LUMC datasets. *CEBPB* expression was increased with advanced cancer stages and in tumors with LN metastasis in both datasets. In addition, *CEBPB* was higher in distal locations and low TMB or MSS (Fig. S8A). Notably, *CEBPB* expression differed significantly according to CMS. CMS2 and CMS4 subtypes showed significantly higher *CEBPB* expression compared with CMS1 and CMS3 in both the TCGA-CRC and Sidra-LUMC datasets (Fig. 4A), suggesting that *CEBPB* overexpression might be related with the immunosuppressive TME in CMS2 and CMS4. We further investigated whether *CEBPB* expression is associated with an immunosuppressive TME by analyzing TIL using Lunit SCOPE IO in the TCGA-CRC dataset. Tumor *CEBPB* expression was inversely associated with T cell infiltration, measured as Inflamed Score in the whole-slide images (Fig. 4B).

As immune-inflamed CRC often shows a favorable prognosis [50], we next evaluated if *CEBPB* expression negatively affects prognosis in CRC. We analyzed PFS and DSS according to *CEBPB* expression among stages I to III in the Sidra-LUMC dataset, which is reported as having a highly curated clinical dataset [41]. *CEBPB*-high patients showed significantly worse PFS (5-year PFS rate: 72.4% vs. 86.4%) and DSS (5-year DSS rate: 82.7% vs. 91.6%) compared with *CEBPB*-low patients (Fig. 4C and Fig. S8B). Moreover, *CEBPB* expression was significantly associated with poor PFS and showed a tendency toward poor DSS after adjusting for clinicopathological covariates in the multivariate analysis (Fig. 4D and Fig. S8C).

### Tumor epithelial cell *CEBPB* and T cell *CTLA4* expression

**Fig. 5.**
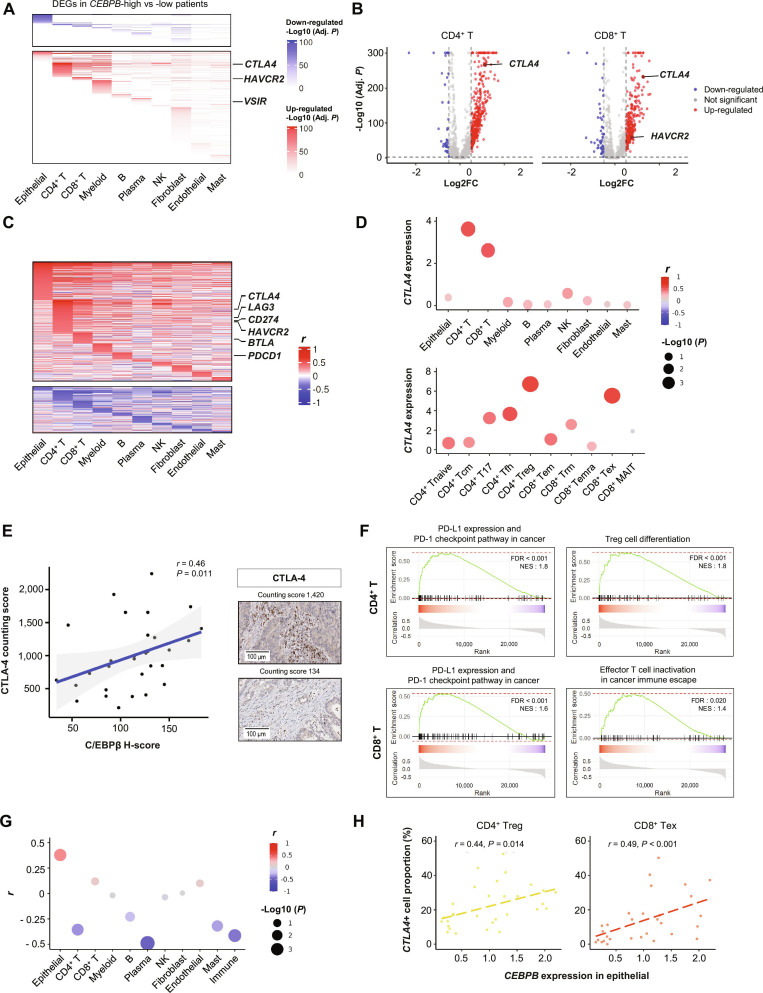
Tumor epithelial cell *CEBPB* expression is associated with T cell *CTLA4* expression. (A) scRNA-seq data of 30 human CRCs were analyzed to determine DEGs between epithelial *CEBPB*-high and -low patient groups across different cell types. (B) Volcano plots showing DEGs in CD4^+^ and CD8^+^ T cells from *CEBPB*-high and -low patient groups. (C) Heatmap of genes in each cell type showing correlation with *CEBPB* expression of epithelial cells. (D) Correlation between epithelial cell *CEBPB* expression and *CTLA4* expression across different cell types (top) and T cell subtypes (bottom). (E) IHC analysis of C/EBPβ and CTLA-4 on human CRC tumor tissues (*n* = 30). Correlation between epithelial cell C/EBPβ H-score and CTLA-4 counting score in TILs was analyzed. Representative images of abundant (counting score 1,420, top right) and scanty (counting score 134, bottom right) CTLA-4^+^ TILs. (F) GSEA of genes in CD4^+^ and CD8^+^ T cells correlated with epithelial cell *CEBPB* expression. (G) Correlation between epithelial cell *CEBPB* expression and the proportion of each cell type. (H) Correlation between epithelial cell *CEBPB* expression and *CTLA4*^+^ cell proportion in CD4^+^ Treg and CD8^+^ Tex. The correlation coefficient (*r*) and *P* value were calculated using Pearson’s correlation analysis. Abbreviations: Adj. *P*, adjusted *P* value; *BTLA*, B- and T-lymphocyte attenuator; *CD274*, cluster of differentiation 274; *CEBPB*, CCAAT enhancer binding protein beta; *CTLA4*, cytotoxic T-lymphocyte associated protein 4; DEG, differentially expressed gene; FDR, false discovery rate; GSEA, gene set enrichment analysis; *HAVCR2*, hepatitis A virus cellular receptor 2; H-score, histoscore; *LAG3*, lymphocyte activating 3; MAIT, mucosal-associated invariant T cell; NES, normalized enrichment score; NK, natural killer cell; PD-1, programmed cell death protein 1; PD-L1, programmed cell death ligand 1; *PDCD1*, programmed cell death 1; Tcm, central memory T cell; Tem, effector memory T cell; Temra, terminally differentiated effector memory T cell; Tex, exhausted T cell; Tfh, T follicular helper cell; TIL, tumor-infiltrating lymphocyte; Tnaive, naive T cell; Trm, tissue-resident memory T cell; Treg, regulatory T cell; *VSIR*, V-set immunoregulatory receptor.

To analyze the effect of tumor epithelial cell *CEBPB* expression on the immune microenvironment, the DEGs of other cell types were analyzed according to the epithelial cell *CEBPB* expression in human scRNA-seq data of 30 CRCs, where high *CEBPB* expression was defined using a GMM (Fig. 5A, Table S7, and Fig. S9A). *CTLA4* was one of the genes simultaneously up-regulated in CD4^+^ and CD8^+^ T cells from tumors with high epithelial cell *CEBPB* expression (Fig. 5B). Next, we analyzed which gene expression in different TME cell types significantly correlated with *CEBPB* expression in epithelial cells using the Pearson correlation analysis. *CTLA4* expression in CD4^+^ and CD8^+^ T cells was positively correlated with epithelial cell *CEBPB* expression, and the association remained significant after adjusting for patient-level clustering in linear mixed-effects models at single-cell resolution (Fig. 5C and D, top, and Fig. S9B). CD4^+^ Treg cells and CD8^+^ Tex cells showed the highest *CTLA4* expression and the strongest correlation with epithelial cell *CEBPB* expression (Fig. 5D, bottom). These associations remained significant even when analyzed exclusively in the MSS patient group (Fig. S9C). Other immune checkpoint genes positively correlated with epithelial cell *CEBPB* expression were *LAG3* and *HAVCR2* in CD4^+^ T cells and B and T lymphocyte attenuator (*BTLA*) in CD8^+^ T cells (Table S8). IHC analysis of human CRC tumor tissues confirmed the positive correlation between C/EBPβ expression in tumor cells and CTLA-4 expression in TILs (Fig. 5E). We also analyzed pathways enriched by the genes in T cells that were positively correlated with *CEBPB* expression in epithelial cells. Genes involved in the “T cell receptor signaling pathway”, “CTLA4 inhibitory signaling”, and “PD-L1 expression and PD-1 checkpoint pathway in cancer” pathways were enriched in CD4^+^ and CD8^+^ T cells (Fig. S9D and Table S9). GSEA confirmed that epithelial cell *CEBPB*-correlated T cell genes were enriched in these pathways, and those additional pathways associated with Treg, such as “Treg cell differentiation” and “Treg cells promote immunosuppression in cancer immune escape”, were also enriched by the genes with a positive correlation in CD4^+^ T cells (Fig. 5F, Fig. S10A, and Table S10).

When we analyzed the relation between epithelial cell *CEBPB* expression and intratumoral cell composition, the proportion of epithelial cells had a positive correlation with epithelial cell *CEBPB* expression, whereas the proportion of immune cells (a combined fraction of CD4^+^ T, CD8^+^ T, myeloid, B, plasma, NK, and mast cells) decreased as epithelial cell *CEBPB* expression increased (Fig. 5G and Fig. S10B). Among immune cells, plasma and CD4^+^ T cells showed prominent negative correlations. However, the proportions of *CTLA4*^+^ (defined using a GMM, Fig. S9A) Treg and *CTLA4*^+^ Tex cells showed positive correlations with *CEBPB* expression in epithelial cells (Fig. 5H). In addition, DEG analysis between *CTLA4*^+^ and *CTLA4*^−^ subpopulations among CD4^+^ Treg cells showed that the *CTLA4*^+^ population was functionally associated with promoting immunosuppression and cancer immune evasion (Fig. S10C). A similar analysis among CD8^+^ Tex cells indicated that *CTLA4*^+^ was linked to impaired effector T cell activity and Notch signaling deregulation (Fig. S10D), which can contribute to immune escape mechanisms.

To validate our observations in an independent dataset, we re-analyzed a publicly available scRNA-seq dataset (GSE178341) of 62 CRC patients. This dataset confirmed the positive correlation between the epithelial cell *CEBPB* expression and *CTLA4* expression in CD4^+^ T, CD8^+^ T, CD4^+^ Treg, and CD8^+^ Tex cells (Fig. S11A and B). Additionally, analysis of spatially resolved transcriptomic datasets from 4 Visium [45] revealed a positive correlation between *CEBPB* and *CTLA4* expression (Fig. S11C and D), indicating spatial co-expression of these 2 genes at the spot level. Notably, the correlation coefficients increased when the spatial resolution was expanded by including the first surrounding layer of each data spot (Fig. S11C). This finding suggested that *CEBPB* and *CTLA4* were co-expressed within a specific range in tumor tissues.

**Fig. 6.**
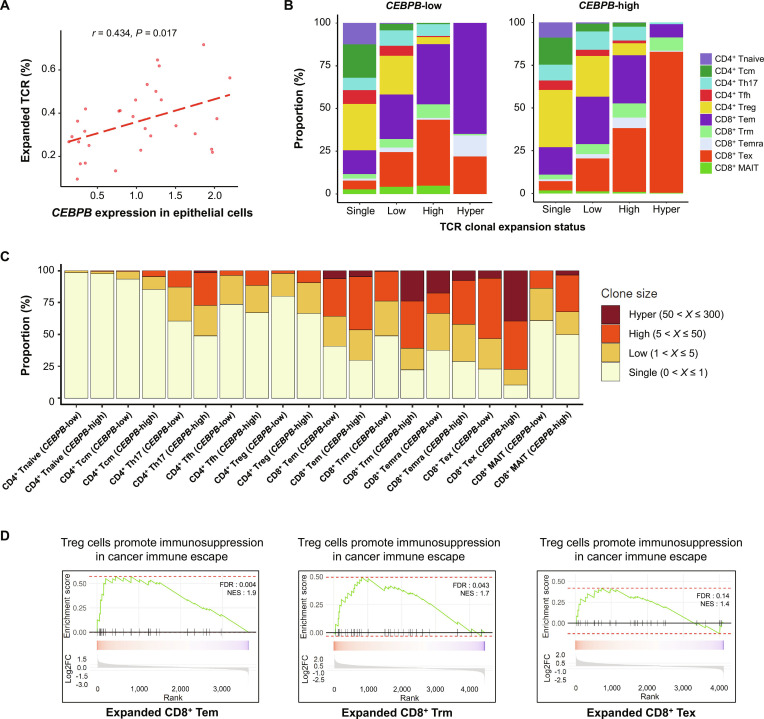
Tumor cell *CEBPB* expression and TCR clonal expansion. (A) Correlation between *CEBPB* expression in epithelial cells and TCR clonal expansion from scRNA-seq of 30 human CRCs. The proportion of T cells with a clonal size of >1 was calculated for each sample. (B) T cell subtype proportions according to TCR clonal expansion status in epithelial cell *CEBPB-*low and -high patient groups. (C) Clonal size distribution across different T cell subtypes. (D) GSEA of DEGs enriched in expanded CD8^+^ Tem (left), CD8^+^ Trm (middle), and CD8^+^ Tex (right) from *CEBPB-*high tumors compared to *CEBPB*-low tumors. The correlation coefficient (*r*) and *P* value were calculated using Pearson’s correlation analysis. Abbreviations: *CEBPB*, CCAAT enhancer binding protein beta; DEG, differentially expressed gene; FDR, false discovery rate; GSEA, gene set enrichment analysis; Log2FC, Log2 fold change; MAIT, mucosal-associated invariant T cell; NES, normalized enrichment score; Tcm, central memory T cell; TCR, T cell receptor; Tem, effector memory T cell; Temra, terminally differentiated effector memory T cell; Tex, exhausted T cell; Tfh, T follicular helper cell; Th17, T helper 17 cell; Tnaive, naive T cell; Trm, tissue-resident memory T cell; Treg, regulatory T cell.

In the analysis of TCR clonal expansion according to epithelial cell *CEBPB* expression in scRNA-seq data of 30 CRCs (Fig. S12A), the proportion of expanded (clone size > 1) TCR clones had a significant correlation with epithelial cell *CEBPB* expression (Fig. 6A). The hyper-expanded clones (clone size > 50) were mostly CD8^+^ Tex in *CEBPB*-high tumors, whereas the majority of hyper-expanded clones in *CEBPB*-low tumors were CD8^+^ effector memory T (Tem) cells (Fig. 6B and Fig. S12B). The increased proportion of highly expanded clones (high and hyper-expanded, clone size > 5) was more prominent in *CEBPB-*high tumors than in *CBBPB*-low tumors in CD8^+^ Tex and CD8^+^ tissue-resident T (Trm) cells (Fig. 6C and Fig. S12B). The gene expression profiles of the expanded CD8^+^ Tem and Trm cells from *CEBPB-*high tumors suggested that these cells were more immunosuppressed than those from *CEBPB-*low tumors (Fig. 6D). In contrast, the *CEBPB*-associated expression profiles in the expanded CD8^+^ Tex cells did not show a significant association with immunosuppression (Fig. 6D).

### Validation of tumor cell *CEBPB* expression affecting T cell *CTLA4* expression

**Fig. 7.**
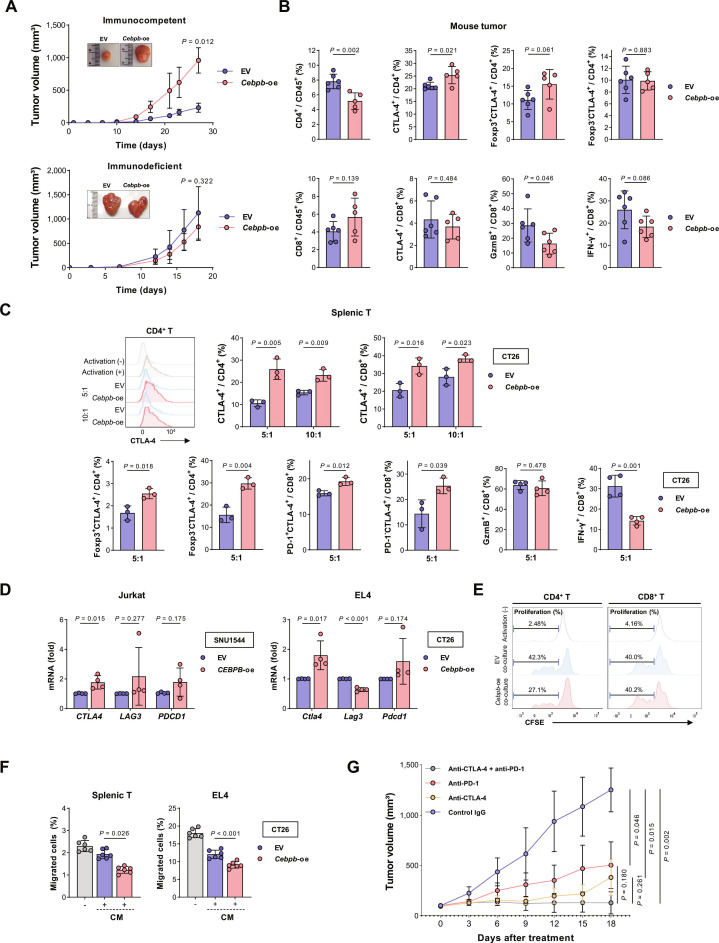
Tumor cell C/EBPβ increases *CTLA-4* expression in CD4^+^ T cells. (A) CT26 cells expressing *Cebpb*-oe and EV control were injected subcutaneously into immunocompetent BALB/c mice (*n* = 4; representative of 3 independent experiments, top) and immunodeficient BALB/c nude mice (*n* = 5; single experiment, bottom). (B) Subcutaneous tumors from EV and *Cebpb*-oe CT26 cells injected into BALB/c mice were collected approximately 21 days post-inoculation. The percentages of CD4^+^ and CD8^+^ cells among CD45^+^ cells, CTLA-4^+^ cells among CD4^+^ and CD8^+^ cells, Foxp3^+^CTLA-4^+^ and Foxp3^−^CTLA-4^+^ cells among CD4^+^ cells, and GzmB^+^ and IFN-γ^+^ cells among CD8^+^ cells were compared by flow cytometry. (C) Mouse splenic T cells isolated from BALB/c mice were activated with anti-CD3ε and anti-CD28, then co-cultured with EV or *Cebpb*-oe CT26 cells for 72 h. The culture ratio of T cells to CT26 cells is indicated. Flow cytometry was used to analyze CTLA-4^+^ and Foxp3^+^ levels among CD4^+^ cells, as well as CTLA-4^+^, PD-1^+^, GzmB^+^, and IFN-γ levels among CD8^+^ cells. Representative histograms of CTLA-4^+^ expression in CD4^+^ T are presented. (D) mRNA expression of immune checkpoint genes in Jurkat (left) and EL4 cells (right) after co-culture with indicated cells, measured by real-time PCR. (E) Proliferation analysis of CFSE-labeled, activated mouse splenic T cells co-cultured with indicated cells for 72 h. CFSE dilution in CD4^+^ T and CD8^+^ T cells were assessed by flow cytometry and presented as histograms (representative of 2 independent experiments). (F) Transwell migration assay of mouse splenic T cells (left) and EL4 (right) cultured for 24 h in CM from *Cebpb*-oe or EV CT26 cells or in fresh media, in the presence of CXCL12 (200 ng/ml). (G) Tumor growth curves of BALB/c mice bearing *Cebpb*-oe CT26 tumors treated with isotype control IgG, anti-PD-1, anti-CTLA-4, or the combination (*n* = 5 per group). Tumor volumes are shown as mean ± SD; statistical comparisons were made using 2-tailed Student’s *t* tests based on the final tumor volume. Bar graphs represent the mean ± SD; each dot represents an independent biological replicate. *P* values were calculated using 2-tailed Student’s *t* tests. Abbreviations: *CEBPB*, CCAAT enhancer binding protein beta; *Cebpb*-oe, *Cebpb*-overexpression; CFSE, carboxyfluorescein succinimidyl ester; CM, conditioned media; CTLA-4, cytotoxic T-lymphocyte associated protein 4; CXCL12, C-X-C motif chemokine ligand 12; EV, empty vector; Foxp3, forkhead box protein P3; *LAG3*, lymphocyte activating 3; PD-1, programmed cell death protein 1; *PDCD1*, programmed cell death 1; SD, standard deviation.

When we subcutaneously implanted *Cebpb*-oe CT26 cells into immunocompetent BALB/c mice, larger tumors were formed compared with EV controls, while no differences in tumor growth were observed in immunodeficient BALB/c nude mice (Fig. 7A and Fig. S13A), and in vitro proliferation and colony formation were also similar between EV and *Cebpb*-oe cells (Fig. S13B). These data suggest that immune evasion may play a role in the enhanced tumor growth by *Cebpb* in immunocompetent mice. In the flow cytometry analysis of the tumors from immunocompetent mice, compared with EV control tumors, *Cebpb*-oe tumors had fewer tumor-infiltrating CD4^+^ T cells, with a higher proportion of CTLA-4^+^ cells and an increasing trend of Foxp3^+^CTLA-4^+^ cells among CD4^+^ T cells, whereas Foxp3^−^CTLA-4^+^ cells remained similar among CD4^+^ T cells (Fig. 7B, top). These in vivo results supported the correlations between tumor cell *CEBPB* expression and CD4^+^ T cell proportion (Fig. 5G) and *CTLA4* expression (Fig. 5D) from human tumor scRNA-seq data. A similar trend was observed in splenic T cells isolated from tumor-bearing mice (Fig. S13C). Regarding CD8^+^ T cells, although CTLA-4 was not increased in CD8^+^ T cells from *Cebpb*-oe tumors, the frequency of effector cytotoxic T cells, defined by granzyme B or interferon-γ expression, was reduced (Fig. 7B, bottom). These findings are consistent with the GSEA results from human CD8^+^ T cells (Fig. 5F).

MC38 cells, which have *Trp53* mutations (G242V and S258I), exhibited higher C/EBPβ expression compared to *Trp53* WT CT26 (Fig. S13A, top). Knockdown of C/EBPβ expression in MC38 using sh*Cebpb* significantly reduced the growth of subcutaneously implanted tumors in the syngeneic immunocompetent C57BL/6N mice (Fig. S13A, bottom). Moreover, mice with sh*Cebpb* MC38 tumors showed an increased CD4^+^ T cell population in the spleen compared to mice bearing shCtrl tumors, while the proportion of Foxp3^+^CTLA-4^+^ subsets within the CD4^+^ T cell population was decreased in the sh*Cebpb* group (Fig. S13D).

We performed in vitro short-term co-culture experiments to validate the observations from scRNA-seq and in vivo data showing that tumor cell *CEBPB* expression could up-regulate T cell *CTLA4* expression. Mouse splenic T cells co-cultured with *Cebpb*-oe CT26 cells demonstrated higher CTLA-4 expression in both CD4^+^ and CD8^+^ T cells than EV controls (Fig. 7C). In this short-term setting, CTLA-4 expression was more broadly induced: both Foxp3^−^ and Foxp3^+^CD4^+^ subsets exhibited increased CTLA-4 expression, and CD8^+^ T cells also up-regulated CTLA-4 irrespective of PD-1 status (Fig. 7C). LAG-3 expression was altered only in the 5:1 ratio condition, whereas PD-1 expression showed no significant change in either the 5:1 or 10:1 ratios (Fig. S13E). CD25 expression was also similar between co-culture with the control and *Cebpb*-oe cells (Fig. S13F), suggesting that the increase in CTLA-4 expression was not a result of additional T cell activation by *Cebpb*-oe cells. Overexpression of *CEBPB* in human CRC cell line SNU1544 or mouse CT26 cell lines also induced *CTLA4* expression in the co-cultured T cell lineage cell lines, human Jurkat and mouse EL4, respectively (Fig. 7D). In contrast, interleukin 2 (*IL2*) expression was not induced by *CEBPB*-oe cells (Fig. S13G), again suggesting that the *CEBPB* overexpression did not activate T cells. Instead, *Cebpb*-oe cells negatively affected T cell proliferation and migration. Co-culture with *Cebpb*-oe CT26 cells decreased the proliferation of splenic CD4^+^ T cells than co-culture with the controls (Fig. 7E). Transwell migration of splenic T cells and EL4 cells using CXCL12 as a chemoattractant was suppressed by CM from *Cebpb*-oe CT26 cells (Fig. 7F and Fig. S13H).

To evaluate the role of CTLA-4 in *Cebpb*-driven immune evasion, BALB/c mice bearing *Cebpb*-oe CT26 tumors were treated with anti-CTLA-4 and anti-PD-1 antibodies. Anti-CTLA-4 monotherapy significantly suppressed tumor growth compared with control IgG treatment, and the combined treatment with anti-PD-1 further reduced tumor growth (Fig. 7G).

### Mediator of tumor cell *CEBPB* and T cell *CTLA4*

**Fig. 8.**
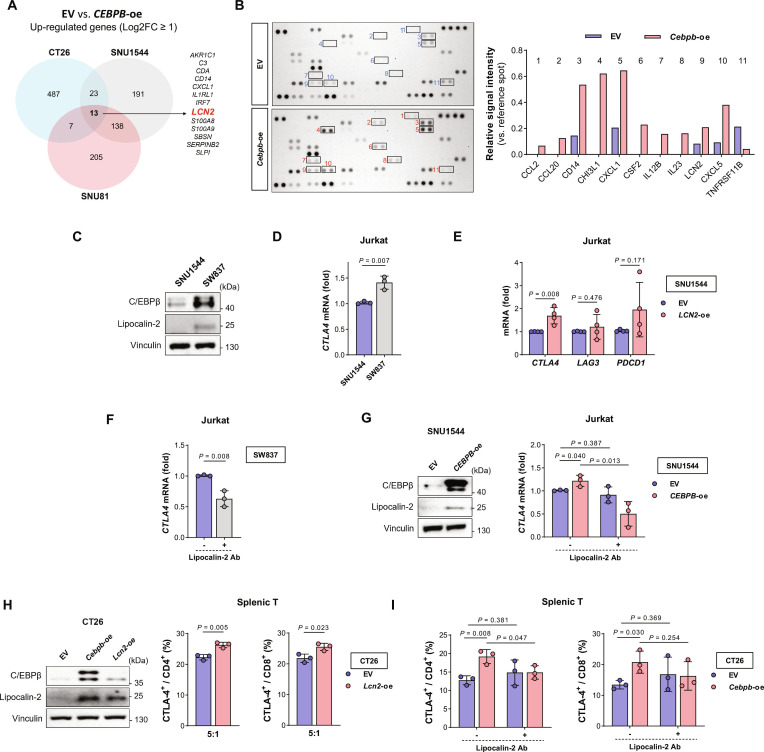
Lipocalin-2 mediates C/EBPβ-driven CTLA-4 up-regulation in T cells. (A) Venn diagram of genes up-regulated in *CEBPB*-oe compared to EV cells in CT26, SNU1544, and SNU81, analyzed by RNA sequencing, identifying 13 genes that were concurrently up-regulated. (B) CM from EV or *Cebpb*-oe CT26 cells were subjected to mouse cytokine array. Each cytokine is represented by duplicate spots, and those showing altered expression are indicated (left). The graph shows signal intensity relative to control spots, analyzed by densitometric quantification (right). (C) Protein levels of C/EBPβ and lipocalin-2 in SNU1544 and SW837 cells by Western blotting*.* (D) mRNA expression of *CTLA4* in Jurkat cells after 24 h co-culture with indicated cells, measured by real-time PCR. (E) mRNA expression of immune checkpoint genes in Jurkat cells after co-culture with *LCN2*-oe or EV SNU1544 cells. (F) mRNA expression of *CTLA4* in Jurkat cells co-cultured with SW837 cells at a 1:1 ratio in the presence of lipocalin-2 neutralizing antibody (1 μg/ml) or isotype IgG control for 24 h. (G) Protein levels of CEBP/β and lipocalin-2 in *CEBPB*-oe and EV SNU1544 cells (left). mRNA levels of *CTLA4* in Jurkat cells co-cultured with *CEBPB*-oe or EV SNU1544 cells in the presence of lipocalin-2 neutralizing antibody (1 μg/ml) or isotype IgG control for 24 h (right). (H) Protein levels of C/EBPβ and lipocalin-2 in EV, *Cebpb*-oe, and *Lcn2*-oe CT26 cells (left). Flow cytometry analysis of CTLA-4^+^ in CD4^+^ and CD8^+^ cells from activated mouse splenic T cells co-cultured with EV or *Lcn*2-oe CT26 cells for 72 h at the indicated culture ratio of T cells to CT26 cells (right). (I) Flow cytometry analysis of activated mouse splenic T cells co-cultured with *Cebpb*-oe CT26 at a 5:1 ratio for 48 h in the presence of lipocalin-2 neutralizing antibody (2 μg/ml) or isotype IgG control. The bar graphs represent the mean ± SD; each dot indicates a biological replicate. *P* values were calculated using 2-tailed Student’s *t* tests. Abbreviations: *AKR1C1*, aldo-keto reductase family 1 member C1; *C3*, complement C3; *CDA*, cytidine deaminase; *CD14*, cluster of differentiation 14; *CEBPB*, CCAAT enhancer binding protein beta; *CEBPB*-oe, *CEBPB* overexpression; CM, conditioned media; *CTLA4*, cytotoxic T-lymphocyte associated protein 4; CXCL1, C-X-C motif chemokine ligand 1; EV, empty vector; *IL1RL1*, interleukin 1 receptor like 1; *IRF7*, interferon regulatory factor 7; *LCN2*, lipocalin-2; *Lcn2*-oe, *Lcn2*-overexpression; *S100A8*, S100 calcium binding protein A8; *S100A9*, S100 calcium binding protein A9; *SBSN*, suprabasin; SD, standard deviation; *SERPINB2*, serpin family B member 2; *SLPI*, secretory leukocyte peptidase inhibitor.

We examined the DEGs from human CRC cell lines SNU81 and SNU1544, as well as the CT26 cell line, following *CEBPB* overexpression to identify the potential mediator between tumor cells and T cells. A total of 13 genes were concurrently up-regulated in all 3 *CEBPB*-oe cell lines (Fig. 8A and Table S11). We also analyzed the secreted proteins in the CM from EV and *Cebpb*-oe CT26 cells using a cytokine array. Of the 13 genes up-regulated by *CEBPB*, 3 encoded proteins (CD14, CXCL1, and LCN2) were included in the cytokine array. They were elevated in the CM from *Cebeb*-oe cells ranging from 2.5- to 3.7-fold (Fig. 8B and Table S12). *LCN2* up-regulation was also confirmed using real-time PCR analysis of the *CEBPB*-oe cell lines (Fig. S14A).

Lipocalin-2 (neutrophil gelatinase-associated lipocalin), encoded by *LCN2*, is a pleiotropic modulator of iron metabolism and inflammation that is elevated in many types of cancer [51]. *LCN2* expression showed a positive correlation with *CEBPB* expression among 80 CRC cell lines in the DepMap mRNA expression database (Fig. S14B), and SW837 and SNU1544 were selected for further experiments. The higher expression levels of C/EBPβ and LCN2 in SW837 compared to SNU1544 were confirmed using Western blotting (Fig. 8C). Co-culture with SW837 cells resulted in higher *CTLA4* expression in Jurkat cells than co-culture with SNU1544 cells (Fig. 8D). We then overexpressed *LCN2* in SNU1544 cells and observed an increase in C*TLA4* expression in co-cultured Jurkat cells (Fig. 8E). Moreover, treatment with lipocalin-2 neutralizing antibody (1 μg/ml) decreased *CTLA4* expression in Jurkat cells co-cultured with SW837 (Fig. 8F) or *CEBPB*-oe SNU1544 cells (Fig. 8G). Similar results were obtained with EL4 cells co-cultured with *Cebpb*-oe CT26 cells (Fig. S14C). These results were confirmed by co-culture experiments using CT26 cells and syngeneic splenic T cells isolated from BALB/c mice. Co-culture with *Lcn2*-oe CT26 cells resulted in increased CTLA-4^+^ expression in CD4^+^ and CD8^+^ T cells compared to co-culture with EV cells (Fig. 8H), whereas the CD25 expression remained unchanged (Fig. S14D). In addition, treatment with the lipocalin-2 neutralizing antibody (2 μg/ml) abrogated CTLA-4 up-regulation in the splenic CD4^+^ T cells driven by *Cebpb*-oe CT26 cells (Fig. 8I).

## Discussion

In the present study, we found that tumor cell *CEBPB* expression in CRC could up-regulate T cell *CTLA4* expression, which may have an important role in immune evasion. Tumor cell *CEBPB* expression also correlated with the T cell expression of genes enriched in the immunosuppressive pathways and clonal expansion of immunosuppressive T cells in the TME. Furthermore, we showed that *CEBPB* expression was negatively associated with TIL infiltration and patients’ survival using publicly available datasets.

Another important finding of this study was that *TP53* negatively regulates *CEBPB* in cancer. *TP53* down-regulated C/EBPβ expression by affecting both mRNA expression and protein stability. Furthermore, the association between *TP53* mutation and *CEBPB* overexpression was confirmed in 2 independent external CRC datasets, and the association was also observed in various other cancers.

Potential mechanisms by which WT *TP53* represses *CEBPB* likely act at multiple levels. At the transcriptional level, p53 can directly bind the *CEBPB* promoter and inhibit transcription [49], and p53 can also antagonize C/EBPβ-dependent transcription [52], thereby limiting its auto-regulation [25]. At the post-transcriptional level, the *CEBPB* 3′-untranslated region has been reported to be targeted by several microRNAs [53,54], and accumulating evidence indicates that p53 regulates microRNA expression [48], suggesting that *CEBPB* mRNA expression could be under indirect control of p53 via a microRNA axis. At the post-translational level, p53 has been shown to repress the deubiquitinase ubiquitin-specific protease 1 (USP1) expression [55], which has been reported to stabilize C/EBPβ by deubiquitination [56]. Collectively, these findings supported a model in which *TP53* restrains *CEBPB* through integrated transcriptional, post-transcriptional, and post-translational mechanisms.

*TP53* regulation of *CEBPB* and subsequent modulation of T cell *CTLA4* expression by tumor cell *CEBPB* adds another communication mechanism between the tumor and microenvironment [8]. Among the several candidate messengers between tumor cells and T cells, previous studies have shown that lipocalin-2 exerts an immunosuppressive effect on the TME and is associated with poor prognosis [51,57,58]. Our in vitro data also suggested that targeting lipocalin-2 could reverse the immunosuppressive TME and merit further exploration as a part of cancer immunotherapy. The detailed mechanism by which secreted lipocalin-2 from tumor cells promotes *CTLA4* expression in T cells should also be investigated.

Given the poor response of MSS CRC to anti-PD-1/PD-L1 treatment, various combination approaches have been explored in clinical trials. However, the strategies to potentiate anti-PD-1/PD-L1 efficacy by adding the MEK inhibitor cobimetinib [59] or the multi-kinase inhibitor lenvatinib [59] failed to improve treatment outcomes. Combinations with additional immune checkpoint inhibitors, such as anti-CTLA-4 antibodies, ipilimumab [15,16] and tremelimumab [17], or anti-LAG-3 antibody relatlimab [60], were also ineffective in MSS CRC. In contrast, novel anti-CTLA-4 antibodies, botensilimab [18] and muzastotug [19], in combination with anti-PD-1 antibodies, balstilimab and pembrolizumab, respectively, elicited objective tumor responses in recent early-phase clinical trials. Moreover, regorafenib, a multi-kinase inhibitor, in combination with ipilimumab and nivolumab (RIN) also showed anti-tumor activity in a phase 1 study [61]. These clinical data suggested that targeting CTLA-4 may be an effective immunotherapeutic strategy for MSS CRC. We believe that the scRNA-seq and preclinical data presented herein provide a strong scientific background for immunotherapy targeting CTLA-4 in MSS CRC. The objective response rates in patients without liver metastasis were 22% for botensilimab and balstilimab [18,61] and 36% for the RIN regimen [61]. Considering the moderate anti-tumor activity observed, immunotherapy strategies incorporating anti-CTLA-4 could be further improved by considering the immune evasion mechanisms of individual tumors, such as *CEBPB* expression.

The main limitation of the present study was that only primary CRC tumors were analyzed in our scRNA-seq analysis and publicly available datasets. It is now widely recognized that CRC liver metastasis responds poorly to immunotherapy compared to other metastatic sites [18,19,61,62]. How tumor cells adapt to various metastatic microenvironments and how different components of the microenvironment collaborate to enhance immune evasion and immunotherapy resistance needs to be elucidated in future studies.

## Conclusion

In conclusion, our investigation into the immunosuppressive TME driven by tumor cells revealed that *CEBPB* expression may have a central role in the immunoregulatory mechanism of CRC by up-regulating T cell *CTLA4* expression. We also showed that *LCN2*, a downstream secretory messenger of *CEBPB*, could be a potential therapeutic target.

## Ethical Approval

This study was approved by the IRB of Seoul National University Hospital (IRB number: 2008-131-1150). All patients provided written informed consent before surgery and the Declaration of Helsinki in biomedical research involving human subjects was followed. The mouse experimental protocol was approved by the IACUC at Seoul National University (IACUC number: SNU-220502-02 and SNU-220502-09) and was performed in accordance with the guidelines of the IACUC.

## Data Availability

All raw FASTQ sequencing data generated in this study—including mouse scRNA-seq, as well as human scRNA-seq, T cell receptor sequencing, and whole-exome sequencing—have been deposited in the publicly accessible Korea BioData Station (KBDS) under accession ID KAP241035 (https://kbds.re.kr/KAP241035). For comparative analyses, we also used the publicly available human colorectal cancer scRNA-seq dataset (https://www.ncbi.nlm.nih.gov/geo/query/acc.cgi?acc=GSE178341) and a Visium spatial-transcriptomics dataset from Zenodo (https://doi.org/10.5281/zenodo.7551712).
