# Supplementary material for: CEBPB Expression in Tumor Cells Drives Immune Evasion in Colorectal Cancer via CTLA4 Up-regulation in T Cells
Source: Cancer Commun (Lond). 2026 Feb 24;46:0013. doi: 10.34133/cancomm.0013 (PMC12929812; doi:10.34133/cancomm.0013)
Supplement: Supplementary 1 — Figs. S1 to S14 Tables S1 to S12 [file cancomm.0013.f1.zip › 25-00380 Supplementary Figures - final.pdf]

***CEBPB* expression in tumor cells drives immune evasion in colorectal cancer via *CTLA4* upregulation in T cells**

Hye Jeong Yun[1,†], Chan Ho Park[2,†], Dahye Yun[1,†], Hye-Ri Shin[3,†], Jeong Dong Lee[1], Changhee Park[4], Kiyeon Kim[2], Heejun Shim[2], Hyejin Sim[1], Se Min Kim[1], Min Jung Kim[5], Ji Won Park[5], Seung-Bum Ryoo[5], Yoojoo Lim[6], Seung-Yong Jeong[5], Kyu Joo Park[5], Tae-You Kim[1,4], Junil Kim[2,7,*], Jae-Kyung Won[3,*], and Sae-Won Han[1,4,*]

[1]Cancer Research Institute, Seoul National University College of Medicine, Seoul, Korea

[2]Department of Bioinformatics, Soongsil University, Seoul, Korea

[3]Department of Pathology, Seoul National University Hospital, Seoul National University College of Medicine, Seoul, Korea

[4]Department of Internal Medicine, Seoul National University Hospital, Seoul, Korea

[5]Department of Surgery, Seoul National University Hospital, Seoul, Korea

[6]Lunit Inc., Seoul, Korea

[7]School of Systems Biomedical Science, Soongsil University, Seoul, Korea

[†]Hye Jeong Yun, Chan Ho Park, Dahye Yun, and Hye-Ri Shin contributed equally to this work.

[*]Corresponding authors:

Sae-Won Han (saewon1@snu.ac.kr)

Jae-Kyung Won (jkwon@snuh.org)

Junil Kim (junilkim@ssu.ac.kr)

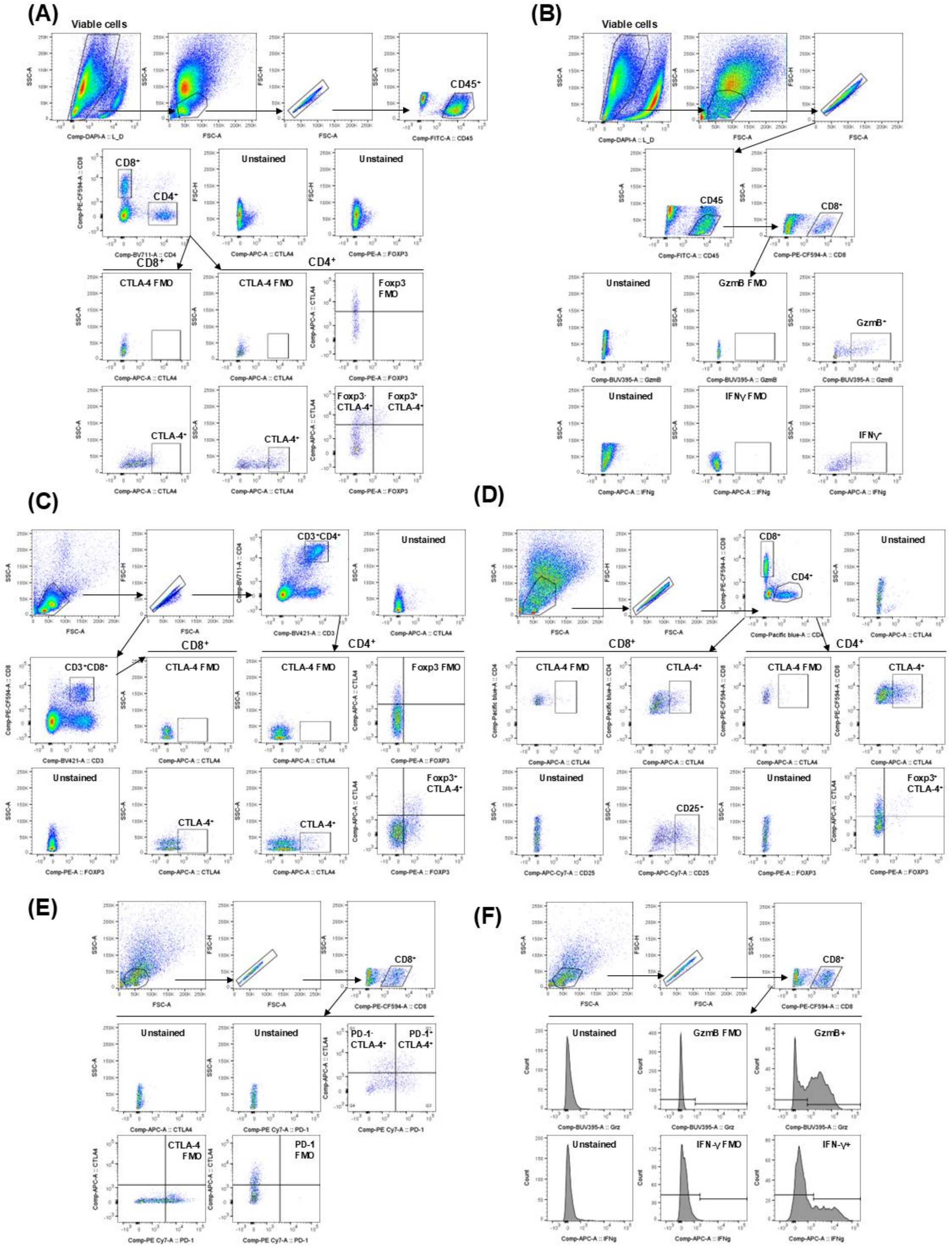

Supplementary Figure S1. **Representative gating strategies used for flow cytometry. (A-B)** Gating strategies used for the experiments shown in **Figure 7B**. Viable cells were first selected for analysis, and cell debris and large aggregates were excluded using FSC and SSC parameters. Immune cell populations (CD45^{+}) were gated and further analyzed for T cell subsets (CD4^{+} and CD8^{+}) and CTLA-4 expression. CD4^{+} T cells were subsequently gated to identify Foxp3^{+}CTLA-4^{+} and Foxp3^{-}CTLA-4^{+} populations **(A)**, and CD8^{+} T cells were further analyzed for GzmB

and IFN-γ expression **(B)**. **(C)** Gating strategies used for the experiments shown in **Supplementary Figure S13C-D**. Spleen cell suspensions isolated from tumor-bearing mice were analyzed to determine the proportions of T cell subsets ($CD3^+CD4^+$ and $CD3^+CD8^+$) and CTLA-4 expression within each subset. $CD4^+$ T cells were subsequently gated to identify $Foxp3^+CTLA\text{-}4^+$ and $Foxp3^-CTLA\text{-}4^+$ populations. **(D-F)** Gating strategies used for the experiments shown in **Figure 7C** and **Supplementary Figure S13E-F**. Mouse splenic T cells co-cultured with CT26 cells for 72 h were analyzed for CTLA-4 and CD25 expression within $CD4^+$ and $CD8^+$ T cell populations, as well as for the $Foxp3^+CTLA\text{-}4^+$ subsets within $CD4^+$ T cells **(D)**, for $PD\text{-}1^+CTLA\text{-}4^+$ subsets within $CD8^+$ T cells **(E)**, for IFN-γ and GzmB expression in $CD8^+$ T cells **(F).** Gating thresholds were determined based on both unstained and FMO controls to define stringent cutoffs.

Abbreviations: CTLA-4, cytotoxic T-lymphocyte associated protein 4; FMO, fluorescence minus one; Foxp3, forkhead box protein P3; FSC, forward scatter; GzmB, granzyme B; IFN-γ, interferon gamma; PD-1, programmed cell death protein 1; SSC, side scatter.

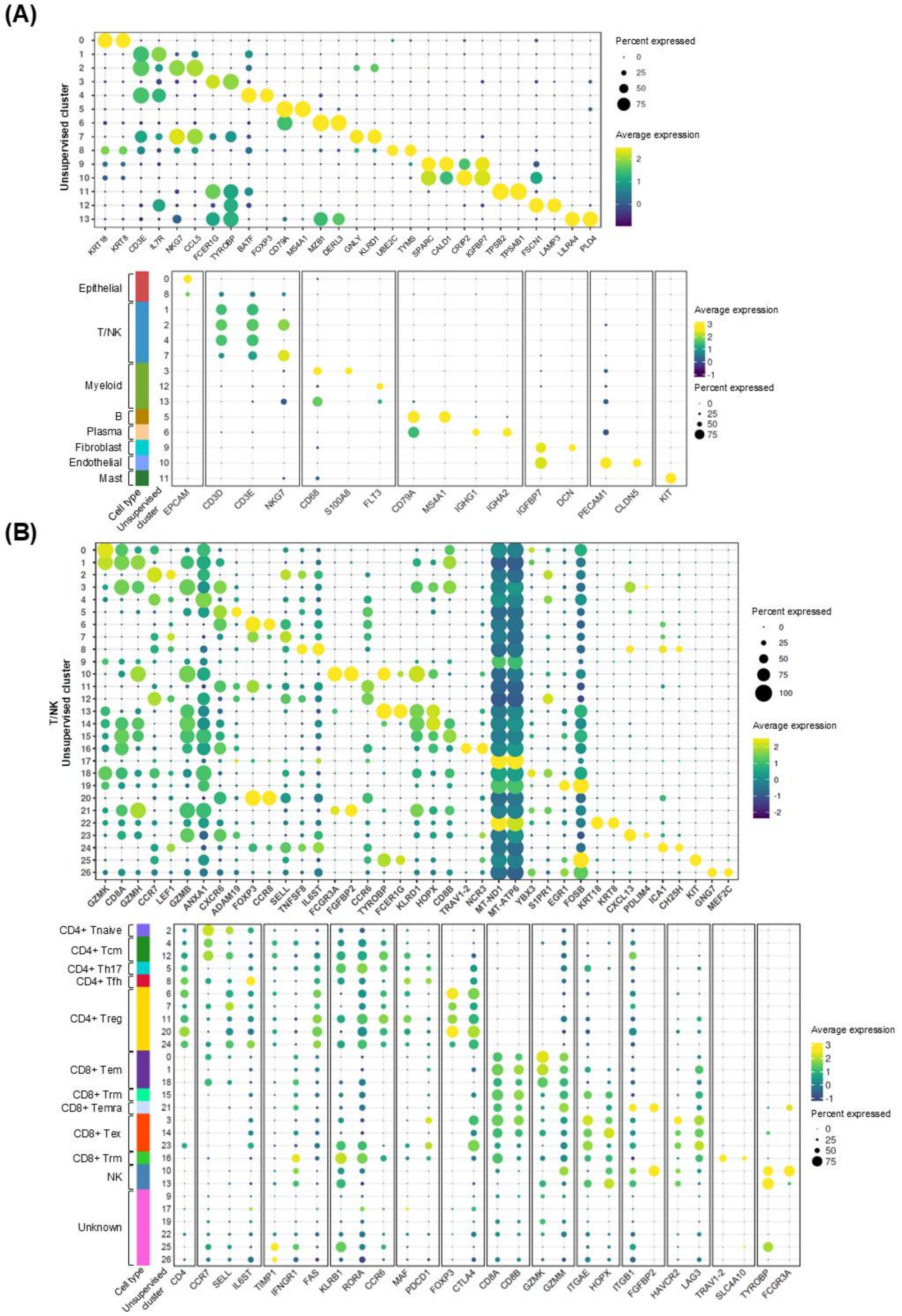

Supplementary Figure S2. Annotation of human CRC TME cell types and T cell subtypes. (A) Dot plot displaying the top two DEGs identified in each graph-based unsupervised cluster from scRNA-seq of tumor tissues from CRC patients (*n* = 30) (top). Dot size indicates the proportion of cells expressing each gene, and color intensity represents average expression. Dot plot showing canonical marker expression across these clusters to annotate cell types (bottom). **(B)** Dot plot showing

the top two DEGs identified in each unsupervised cluster within the T/NK cell (top). Dot plot displays the expression of markers used for annotating T/NK cell subtypes (bottom).

Abbreviations: CRC, colorectal cancer; DEG, differentially expressed gene; MAIT, mucosal-associated invariant T cell; scRNA-seq, single-cell RNA sequencing; T/NK, T cell/natural killer cell; Tcm, central memory T cell; Tem, effector memory T cell; Temra, terminally differentiated effector memory T cell; Tex, exhausted T cell; Tfh, T follicular helper cell; Th17, T helper 17 cell; TME, tumor microenvironment; Tnaive, naive T cell; Trm, tissue-resident memory T cell; Treg, regulatory T cell.

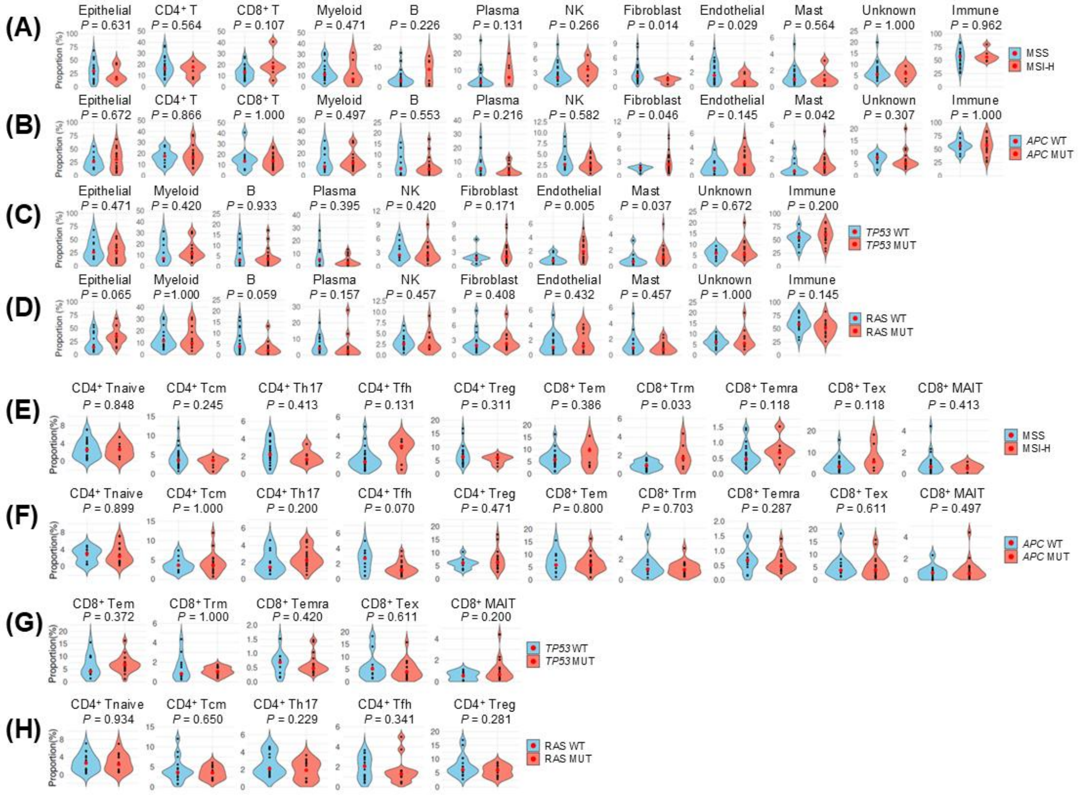

Supplementary Figure S3. Proportions of human CRC TME cell types and T cell subtypes according to mutation status. (A-D) Violin plots showing the proportions of each cell type in scRNA-seq data of 30 CRCs according to MSI status **(A)**, and mutation status of *APC* **(B)**, *TP53* **(C)**, and RAS **(D)**. Violin plots already shown in **Figure 1D** are not duplicated here. **(E-H)** Violon plots showing the proportions of each T cell subtype according to MSI status **(E)**, and mutation status of *APC* **(F)**, *TP53* **(G)**, and RAS **(H)**. Red dots indicate the median. Statistical comparisons were performed using the Wilcoxon rank-sum test.

Abbreviations: *APC*, adenomatosis polyposis coli; CRC, colorectal cancer; MAIT, mucosal-associated invariant T cell; MSS, microsatellite stable; MSI, microsatellite instability; MSI-H, microsatellite instability-high; MUT, mutation; NK, natural killer cell; RAS, rat sarcoma virus; Tcm, central memory T cell; Tem, effector memory T cell; Temra, terminally differentiated effector memory T cell; Tex, exhausted T cell; Tfh, T follicular helper cell; Th17, T helper 17 cell; TME, tumor microenvironment; Tnaive, naive T cell; Treg, regulatory T cell; Trm, tissue-resident memory T cell; *TP53*, tumor protein 53; WT, wild-type.

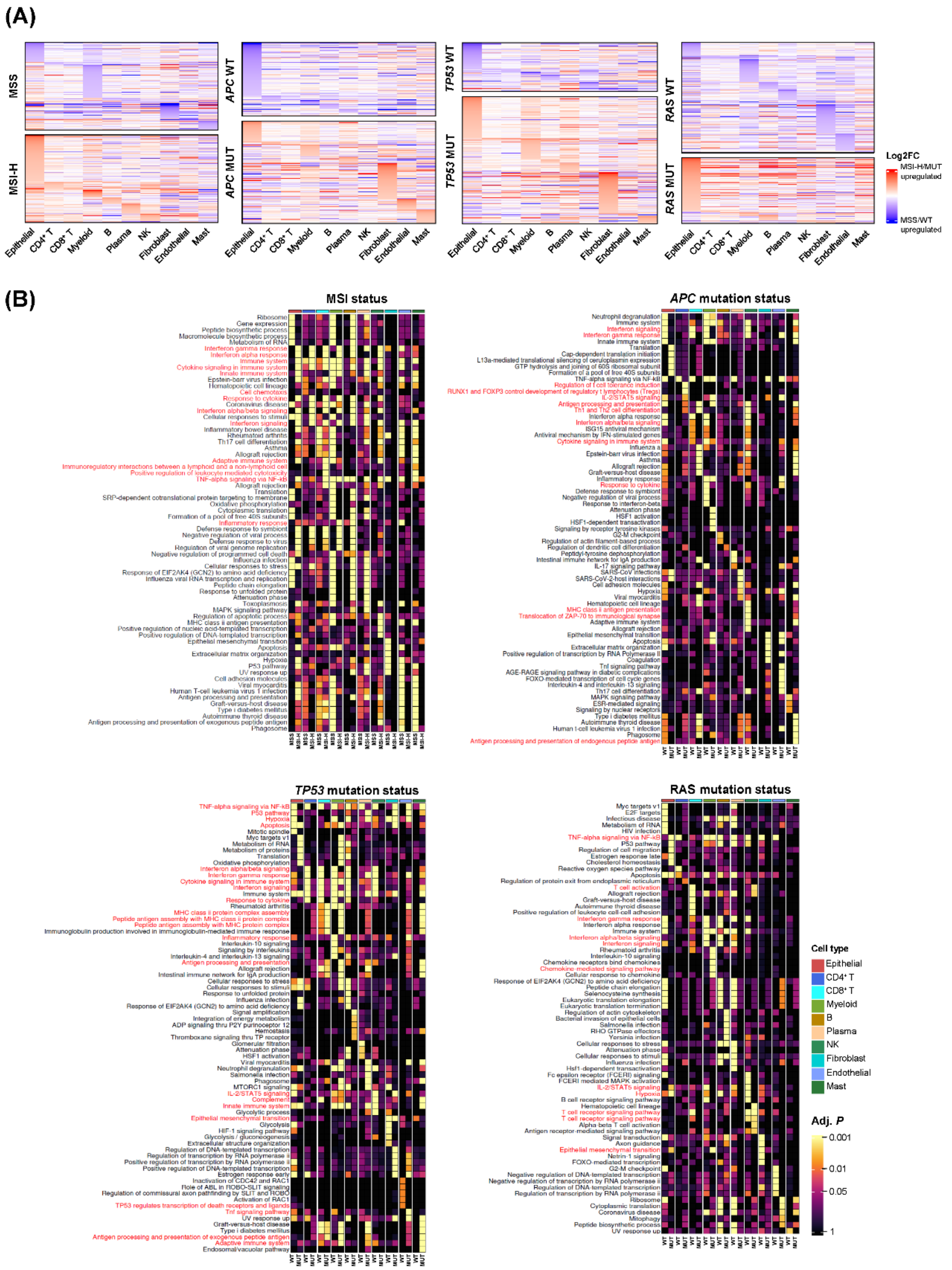

Supplementary Figure S4. Cell type-specific DEG and pathway analysis according to MSI and mutation status. (A) Expression of DEGs across different cell types identified from scRNA-seq data of 30 CRCs, comparing MSI-H and MSS status, as well as WT and MUT status of *APC*, *TP53*, and RAS. Genes with Log2FC ≥ 0.5 and adj. *P* < 0.05 were classified as significant. **(B)** Pathway enrichment analysis for DEGs identified between different genotypes in (A) was performed using EnrichR (databases: KEGG, GO Biological Process, MSigDB, and Reactome). The top five

terms for each cell type are presented. Immune-associated pathways are highlighted in red.

Abbreviations: Adj. *P*, adjusted *P* value; *APC*, adenomatosis polyposis coli; CRC, colorectal cancer; DEG, differentially expressed gene; GO, Gene Ontology; KEGG, Kyoto Encyclopedia of Genes and Genomes; Log2FC, Log2 fold change; MAIT, mucosal-associated invariant T cell; MSI, microsatellite instable; MSI-H, microsatellite instability-high; MSS, microsatellite stable; MUT, mutation; NK, natural killer cell; RAS, rat sarcoma virus; scRNA-seq, single-cell RNA sequencing; *TP53*, tumor protein 53; WT, wild-type.

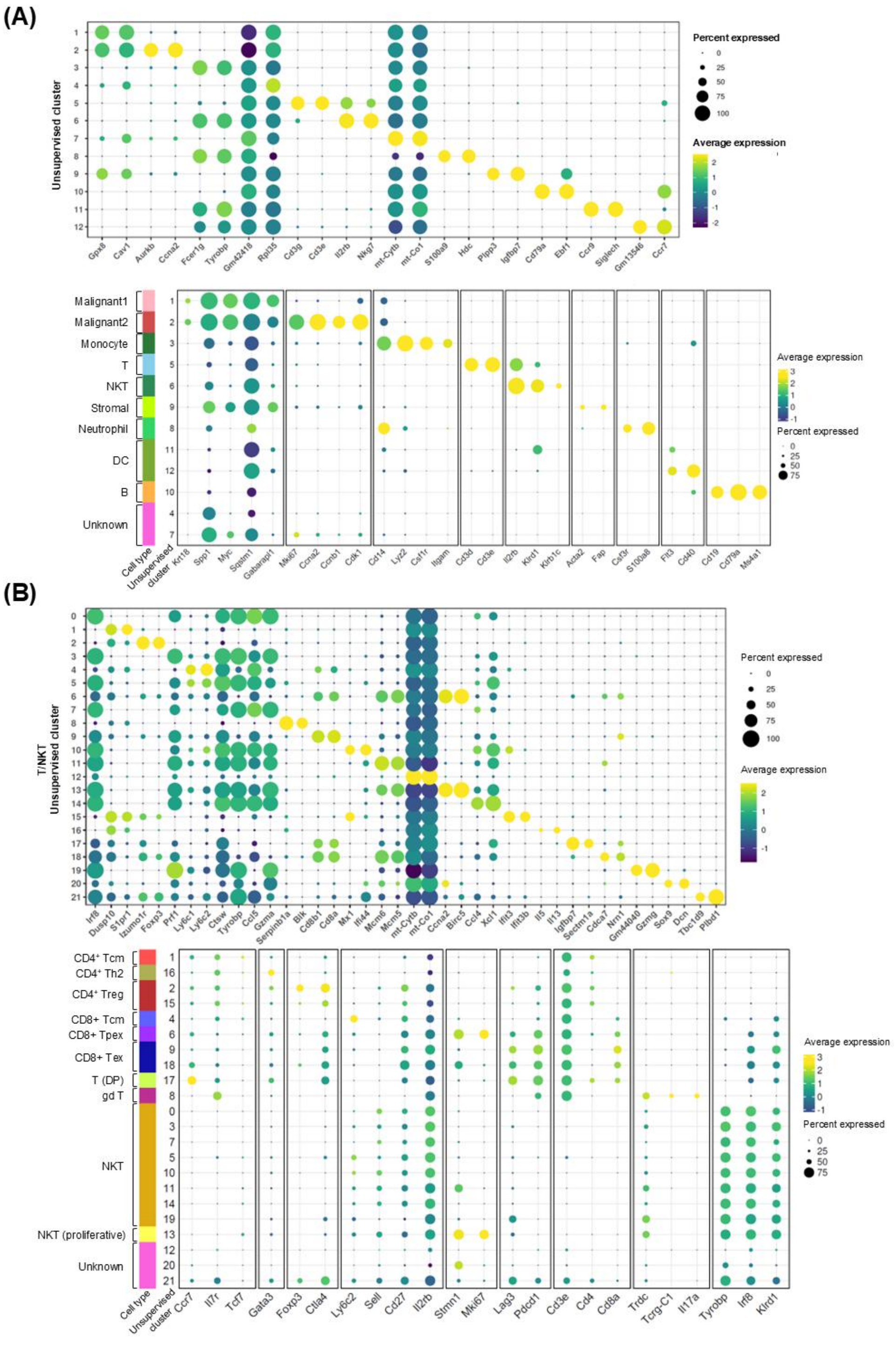

Supplementary Figure S5. Annotation of TME cell types and T cell subtypes of mouse tumor scRNA-seq data. (A) scRNA-seq was performed on mouse tumors grown after subcutaneous injection of *Trp53* KO CT26 and control CT26 cells into syngeneic BALB/c mice, 14 days post-inoculation (n = 2). Dot plot displays the top two DEGs identified in unsupervised clusters of main cell types (top). Dot size indicates the proportion of cells expressing each gene, and color intensity represents

average expression. Dot plot shows the expression of canonical markers to annotate main cell types (bottom). **(B)** Dot plot showing the top two DEGs identified for each unsupervised cluster within the T/NKT cells (top). The dot plot displays the expression of markers used for annotating T/NKT cell subtypes (bottom).

Abbreviations: DC, dendritic cell; DEG, differentially expressed gene; gd T, gamma delta T cell; NKT, natural killer T cell; T (DP), double-positive T cell; T/NK, T cell/natural killer cell; T/NKT, T cell/natural killer T cell; Tcm, central memory T cell; Tex, exhausted T cell; Th2, helper 2 T cell; Tpex, progenitor exhausted T cell; Treg, regulatory T cell.

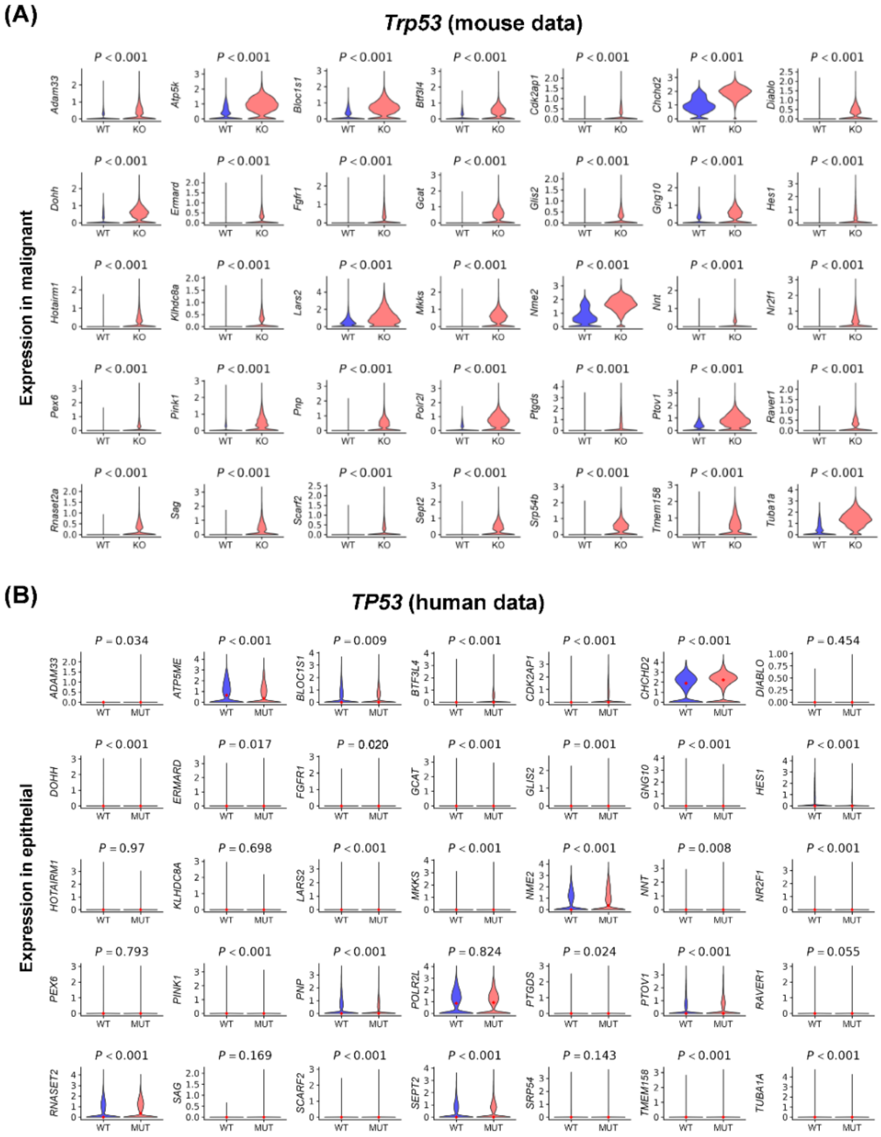

Supplementary Figure S6. *Trp53* mutation-associated gene expression in mouse and human scRNA-seq datasets. (A) The DEGs identified as upregulated genes in both Malignant 1 and 2 cell types in *Trp53* KO tumors compared with control (*Trp53* WT) tumors (**Figure 2D**). **(B)** The expression levels of these DEGs were compared between *TP53* mutation and *TP53* WT human epithelial cells in scRNA-seq data of human 30 CRCs. Statistical comparisons were performed using the Wilcoxon rank-sum test.

Abbreviations: DEG, differentially expressed gene; KO, knockout; MUT, mutation; *TP53*, tumor protein 53; *Trp53*, transformation related protein 53; *Trp53* KO, *Trp53* knockout; WT, wild-type.

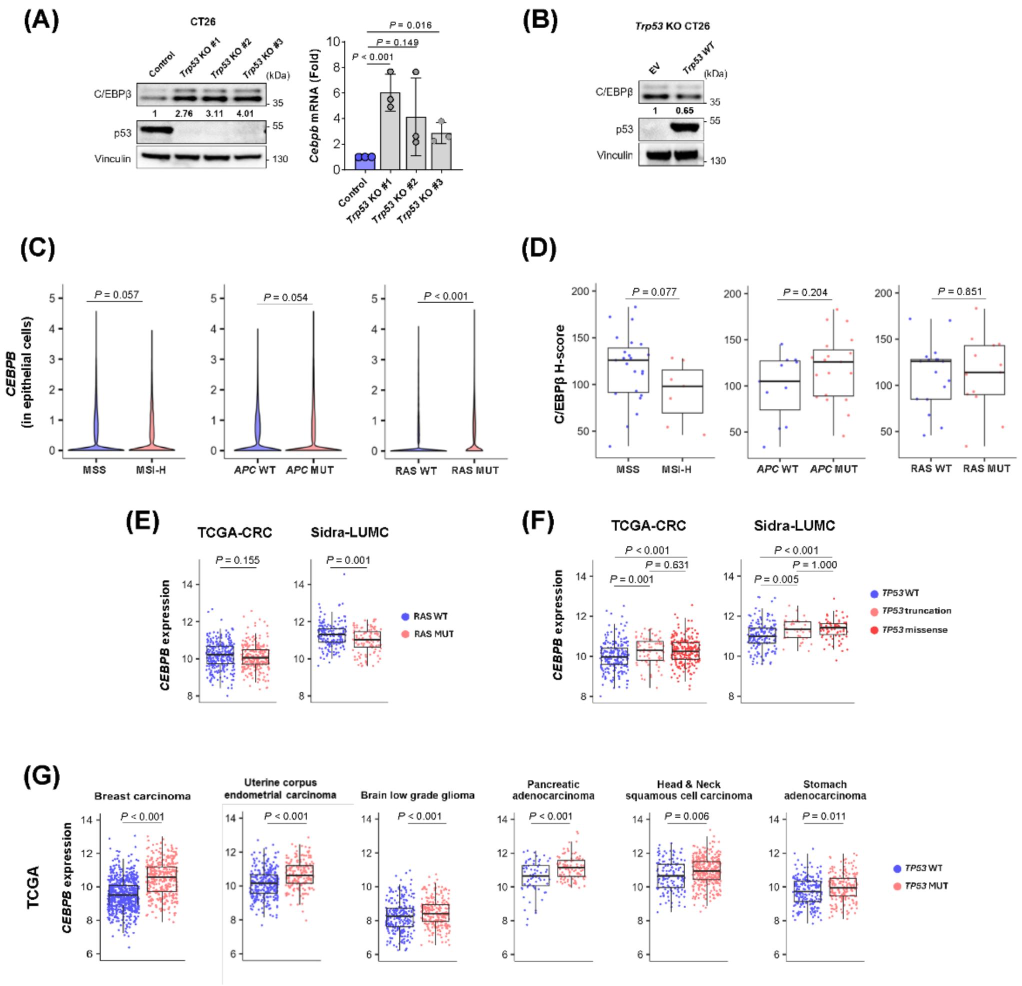

Supplementary Figure S7. ***TP53*** **mutation is associated with elevated** ***CEBPB*** **expression. (A)** Western blotting (left) and real-time PCR (right) results showing *CEBPB* expression levels in 3 independent clones of *Trp53* KO CT26 cells. Values presented underneath the band were generated by densitometric analysis. **(B)** Western blotting showing C/EBPβ expression level following lentiviral expression of *Trp53* WT in *Trp53* KO CT26 cells compared to EV transduced cells. **(C)** Violin plots showing *CEBPB* transcript levels in human epithelial single cells from scRNA-seq data of 30 CRCs according to MSI status and *APC* and RAS mutations. **(D)** Box plots showing H-scores from C/EBPβ IHC in tumor tissues from 30 CRC patients, according to MSI status [MSS (n = 23) vs. MSI-H (n = 7)] and *APC* [WT (n = 11) vs. MUT (n = 19)] and RAS mutations [WT (n = 17) vs. MUT (n = 13)].

(E) Box plots displaying *CEBPB* mRNA levels in TCGA-CRC (left) and Sidra-LUMC datasets (right) according to RAS mutation status. **(F)** Box plots displaying *CEBPB* mRNA levels in TCGA-CRC (left) and Sidra-LUMC datasets (right),

categorized by the type of *TP53* mutations. Truncating mutation includes nonsense, frameshift insertion, and frameshift deletion. **(G)** *CEBPB* mRNA levels according to *TP53* mutation status across various cancer types from the TCGA dataset. Box plots show upper and lower quartiles, median values as center lines and whiskers extend to 1.5 × interquartile range. *P* values were calculated using the Wilcoxon rank-sum test. Abbreviations: *APC*, adenomatosis polyposis coli; *CEBPB*, CCAAT enhancer binding protein beta; EV, empty vector; H-score, histoscore; IHC, immunohistochemistry; MSI, microsatellite instability; MSI-H, microsatellite instability-high; MSS, microsatellite stable; MUT, mutation; RAS, rat sarcoma virus; real-time PCR, real-time polymerase chain reaction; Sidra-LUMC, Sidra-Leiden University Medical Center; TCGA-CRC, The Cancer Genome Atlas colorectal cancer; *TP53*, tumor protein 53; *Trp53*, transformation related protein 53; *Trp53* KO, *Trp53* knockout; WT, wild-type.

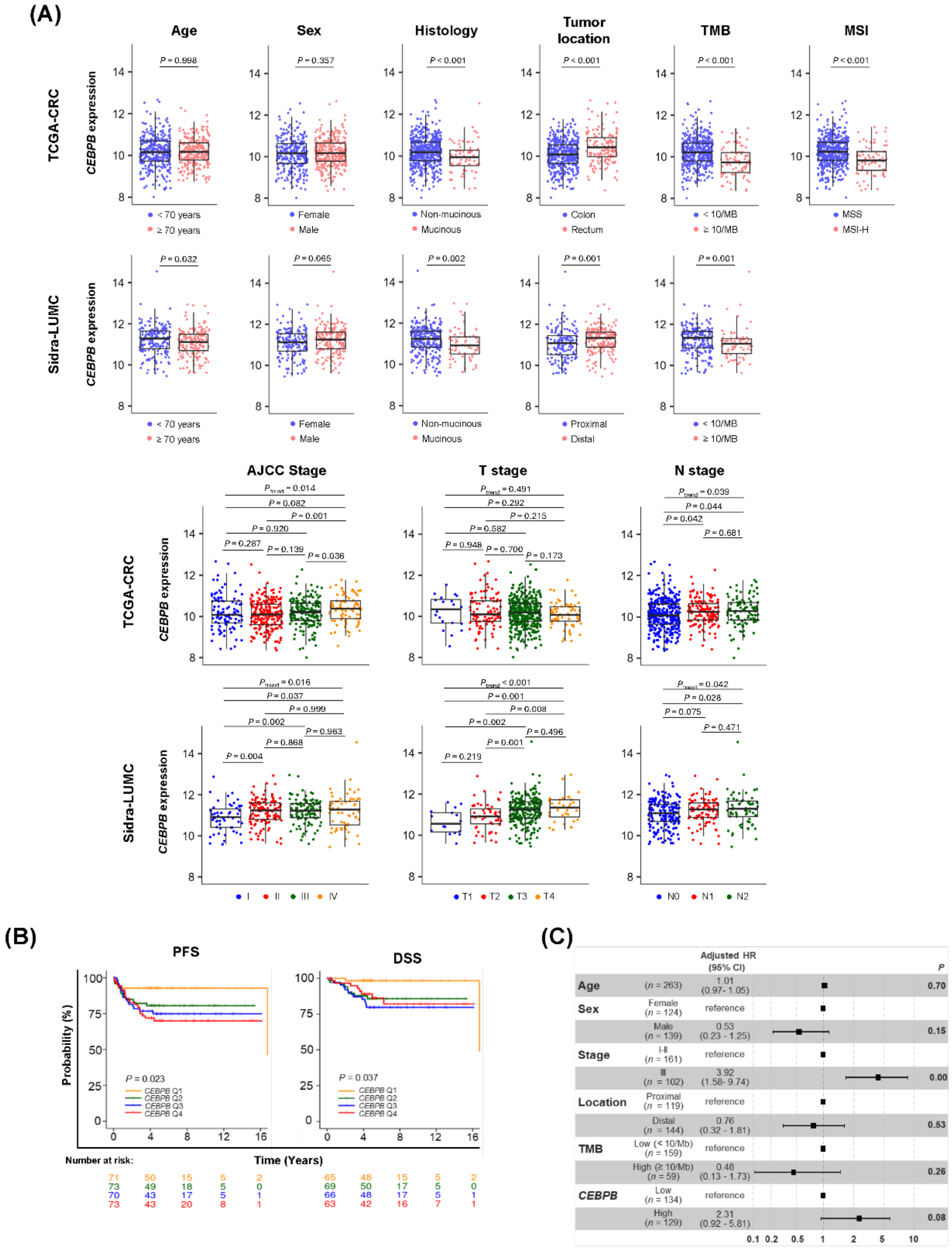

Supplementary Figure S8. Clinicopathological data analysis according to *CEBPB* expression (A) Box plots of *CEBPB* mRNA expression showing the comparison by age, sex, histology, tumor location, TMB, MSI (Sidra-LUMC data not available), AJCC stage, T stage, and N stage in the TCGA-CRC and Sidra-LUMC datasets. Box plots show upper and lower quartiles, median values as center lines and whiskers extend to 1.5 × interquartile range. *P* value for trend was calculated using the Kruskal-

Wallis test. All other *P* values were calculated using the Wilcoxon rank-sum test. **(B)** The Kaplan-Meier plots showing PFS and DSS according to quartile groups of *CEBPB* expression in the Sidra-LUMC dataset. *P* values were calculated using log-rank tests. **(C)** Forest plots presenting the adjusted HR and 95% CI from multivariate analysis of DSS using a Cox proportional hazard model.

Abbreviations: AJCC, American Joint Committee on Cancer; *CEBPB*, CCAAT enhancer binding protein beta; CI, confidence interval; DSS, disease-specific survival; HR, hazard ratio; Mb, megabase; MSI, microsatellite instability; MSI-H, microsatellite instability-high; MSS, microsatellite stable; N, lymph node; PFS, progression-free survival; Sidra-LUMC, Sidra-Leiden University Medical Center; T, tumor; TCGA-CRC, The Cancer Genome Atlas colorectal cancer; TMB, tumor mutational burden.

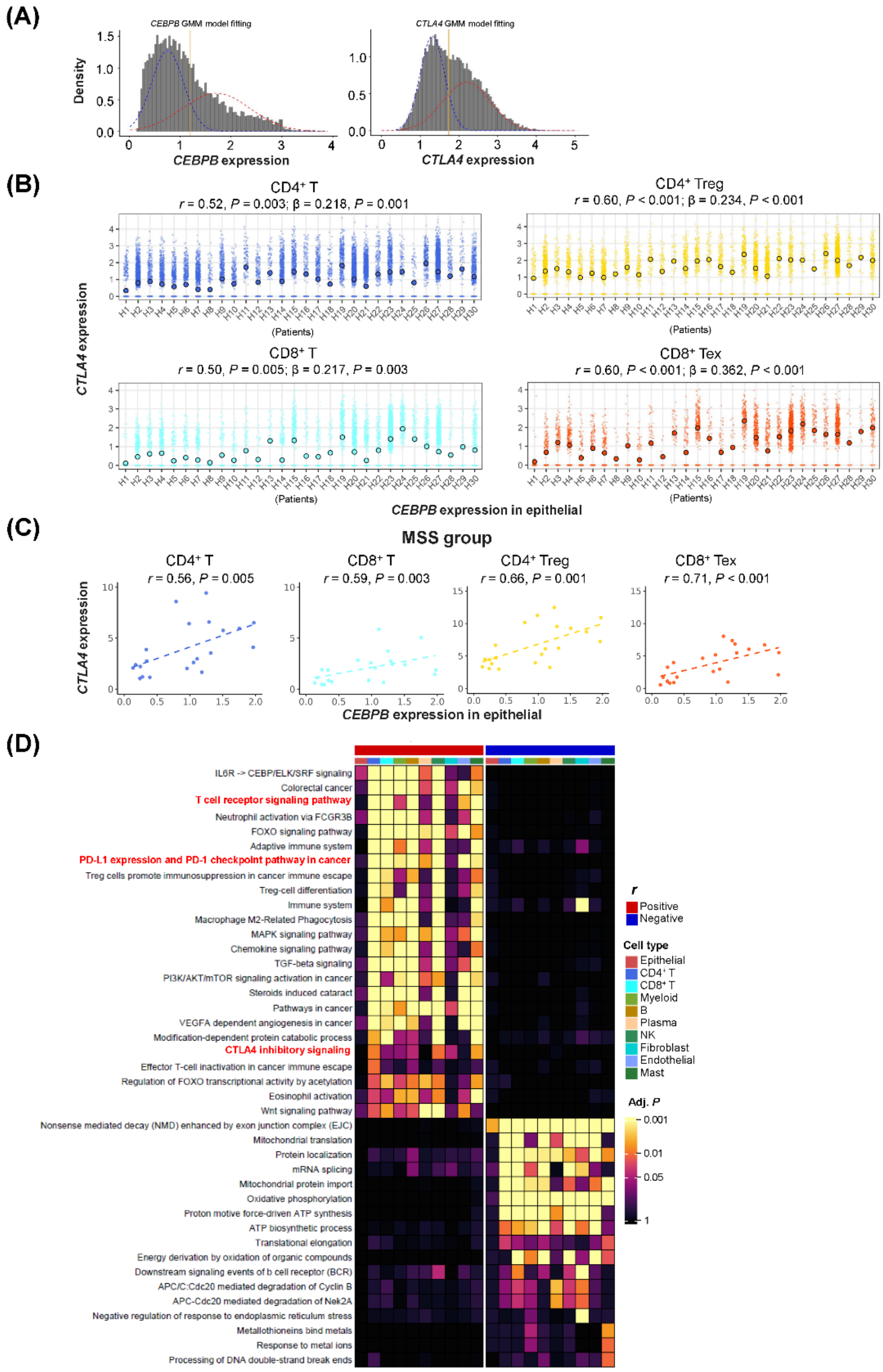

Supplementary Figure S9. *CTLA4* expression in T cell subsets and pathway enrichment analysis of genes associated with epithelial *CEBPB* levels. (A) *CEBPB* expression in epithelial cells (left) and CTLA4 expression in T cells (right) from scRNA-seq data of 30 CRCs, each divided into high and low groups based on a GMM. The threshold for grouping is set at the intersection of two fitted distributions (gold color vertical line). **(B)** Associations between *CTLA4* expression in the indicated cell

types and epithelial *CEBPB* across 30 CRCs. Patient-level PCC (*r*, *P*) and single-cell linear mixed-effects estimates accounting for patient clustering (β, *P*) are shown; each point denotes one patient **(C)** *CTLA4* expression in the indicated T cell subtypes and *CEBPB* expression in epithelial cells were analyzed with Pearson correlation in the MSS patient subgroup ($n = 23$) from scRNA-seq data of 30 CRCs. **(D)** Genes from each cell type meeting the criteria of PCC ($r > 0.25$, $P < 0.05$) in the correlation analysis with average epithelial *CEBPB* expression were subjected to pathway enrichment analysis and are presented in the heatmap.

Abbreviations: Adj. *P*, adjusted *P* value; *CEBPB*, CCAAT enhancer binding protein beta; CRC, colorectal cancer; *CTLA4*, cytotoxic T-lymphocyte associated protein 4; GMM, Gaussian mixture model; MSS, microsatellite stable; NK, natural killer cell; PCC, Pearson’s correlation coefficient; scRNA-seq, single-cell RNA sequencing; Tex, exhausted T cell; Treg, regulatory T cell.

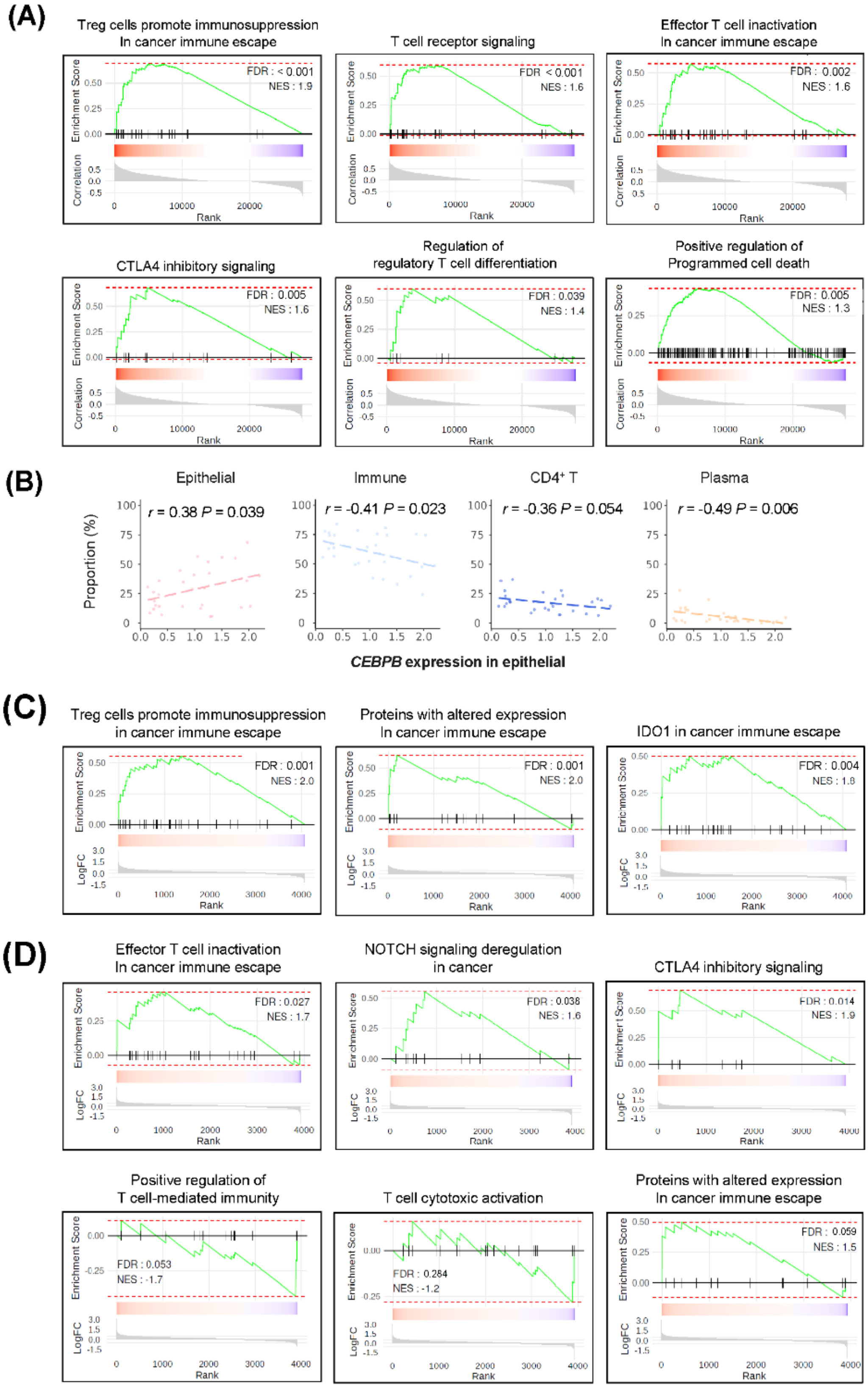
(A)
Treg cells promote immunosuppression
In cancer immune escape
FDR : < 0.001
NES : 1.9
T cell receptor signaling
FDR < 0.001
NES : 1.6
Effector T cell inactivation
In cancer immune escape
FDR : 0.002
NES : 1.6
CTLA4 inhibitory signaling
FDR : 0.005
NES : 1.6
Regulation of
regulatory T cell differentiation
FDR : 0.039
NES : 1.4
Positive regulation of
Programmed cell death
FDR : 0.005
NES : 1.3
Enrichment Score
Correlation
Rank
(B)
Epithelial
r = 0.38 P = 0.039
Immune
r = -0.41 P = 0.023
CD4+ T
r = -0.36 P = 0.054
Plasma
r = -0.49 P = 0.006
Proportion (%)
CEBPB expression in epithelial
(C)
Treg cells promote immunosuppression
in cancer immune escape
FDR : 0.001
NES : 2.0
Proteins with altered expression
In cancer immune escape
FDR : 0.001
NES : 2.0
IDO1 in cancer immune escape
FDR : 0.004
NES : 1.8
Enrichment Score
LogFC
Rank
(D)
Effector T cell inactivation
In cancer immune escape
FDR : 0.027
NES : 1.7
NOTCH signaling deregulation
in cancer
FDR : 0.038
NES : 1.6
CTLA4 inhibitory signaling
FDR : 0.014
NES : 1.9
Positive regulation of
T cell-mediated immunity
FDR : 0.053
NES : -1.7
T cell cytotoxic activation
FDR : 0.284
NES : -1.2
Proteins with altered expression
In cancer immune escape
FDR : 0.059
NES : 1.5
Enrichment Score
LogFC
Rank

Supplementary Figure S10. Gene set enrichment and correlation analyses of epithelial *CEBPB*-associated T cell profiles. (A) GSEA of CD4$^+$ T cell genes with positive correlations with epithelial *CEBPB* expression in the PCC analysis ($r > 0.25$, $P < 0.05$) derived from scRNA-seq data of 30 CRCs. FDR and NES are shown to indicate pathway significance. **(B)** Correlation analysis of the proportion of indicated cell types and epithelial *CEBPB* expression. **(C-D)** GSEA of DEGs in *CTLA4*-high versus *CTLA4*-low cells within CD4$^+$ Tregs **(C)** and CD8$^+$ Tex **(D)**, with *CTLA4* groups defined as in **Supplementary Figure S9A**.

Abbreviations: *CEBPB*, CCAAT enhancer binding protein beta; *CTLA4*, cytotoxic T-lymphocyte associated protein 4; DEGs, differentially expressed genes; FDR, false discovery rate; GSEA, gene-set enrichment analysis; Log2FC, Log2 fold change; NES, normalized enrichment score; PCC, Pearson's correlation coefficient; Tex, exhausted T cell; Treg, regulatory T cell.

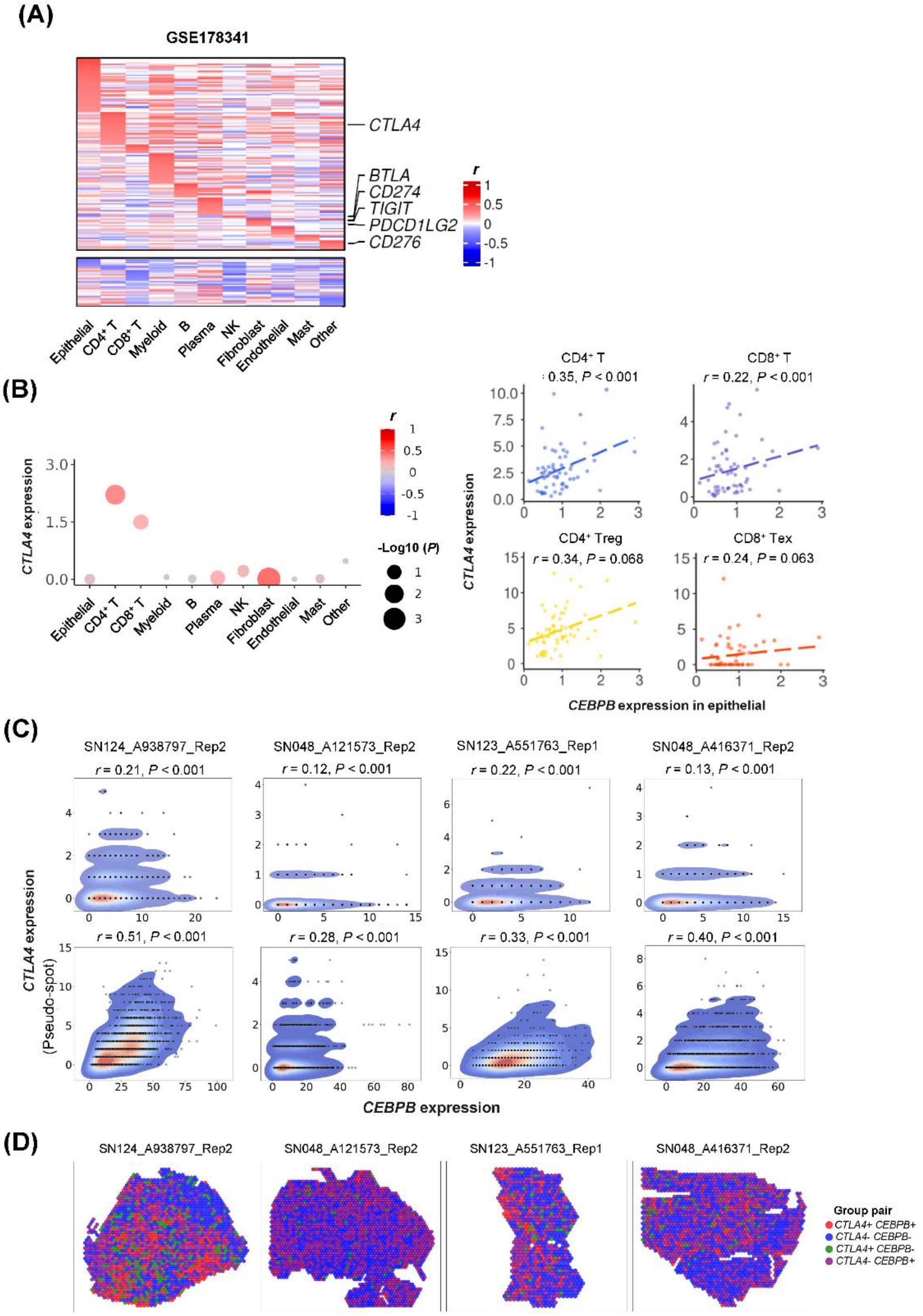

Supplementary Figure S11. Tumor cell *CEBPB* expression and T cell *CTLA4* expression in public datasets of CRC patients. (A-B) Analysis of the public human CRC scRNA-seq dataset (GSE178341, n = 62) showing cell-type–specific DEGs and the correlation between *CTLA4* expression in T cell subtypes and *CEBPB* expression in epithelial cells, as assessed by PCC analysis ($r > 0.25$, $P < 0.05$) **(A)**, correlation of epithelial *CEBPB* expression and *CTLA4* expression across various cell types (left) and in CD4$^+$ T, CD8$^+$ T, CD4$^+$ Treg, and CD8$^+$ Tex (right), as assessed by PCC

analysis **(B)**. **(C-D)** Correlation between *CEBPB* and *CTLA4* expression was analyzed in public spatial transcriptome RNA-seq datasets of four Visium slides from CRC patients. PCC analysis of *CEBPB* and *CTLA4* expression with *CTLA4*-expressing spots exceeding 5% **(C,** top**)**, and to extend the range of PCC calculation, pseudo-spot data were analyzed **(C,** bottom**)**; spatial distribution of spots classified into four groups based on *CTLA4* and *CEBPB* expression levels across the Visium slides **(D)**. Abbreviations: *BTLA*, B- and T-lymphocyte attenuator; *CD274,* cluster of differentiation 274; *CD276*, cluster of differentiation 276; *CEBPB*, CCAAT enhancer binding protein beta; CRC, colorectal cancer; *CTLA4*, cytotoxic T-lymphocyte associated protein 4; DEG, differentially expressed gene; NK, natural killer cell; PCC, Pearson's correlation coefficient; *PDCD1LG2*, programmed cell death 1 ligand 2 (PD-L2); RNA-seq, RNA sequencing; Tex, exhausted T cells; *TIGIT*, T cell immunoreceptor with Ig and ITIM domains; Treg, regulatory T cells.

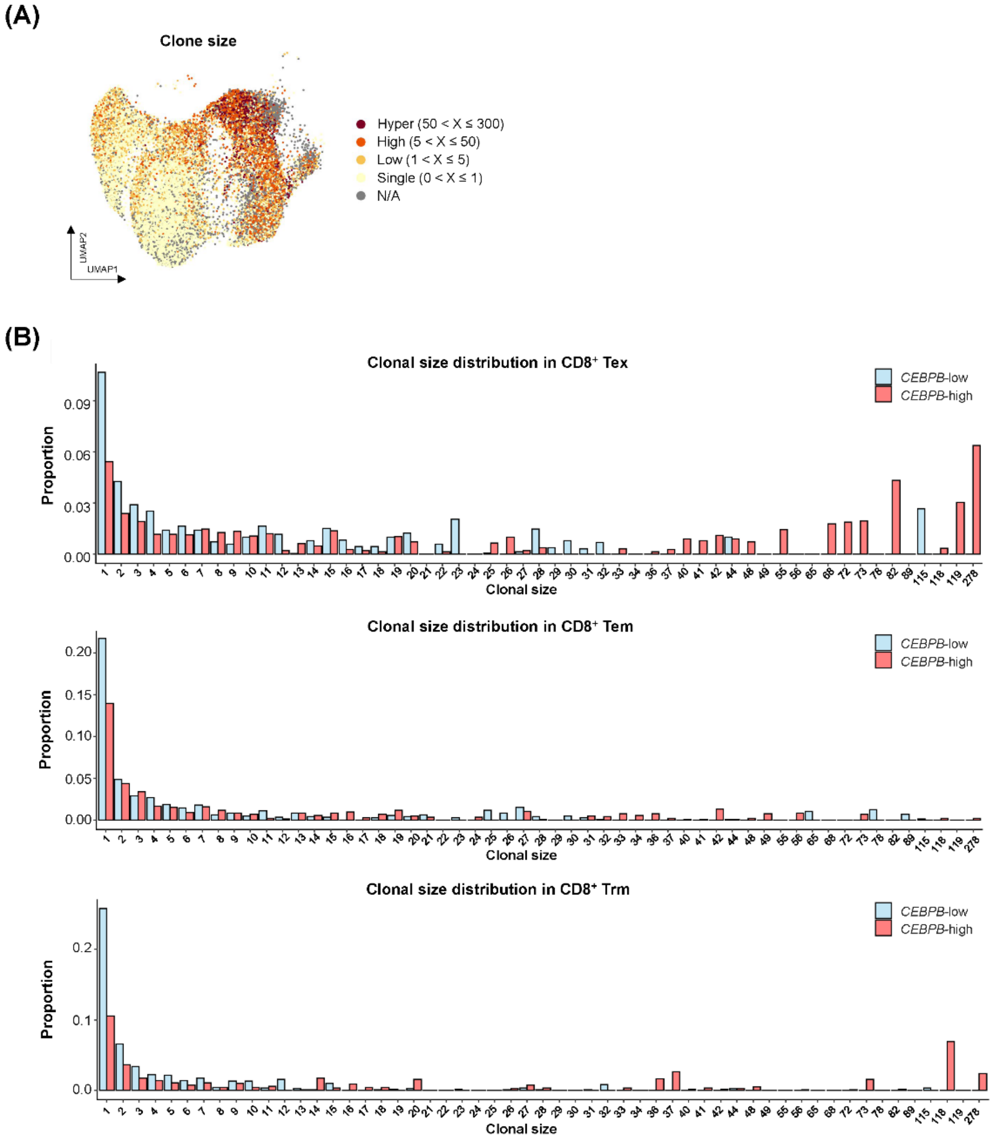

Supplementary Figure S12. Tumor cell *CEBPB* expression and TCR clonal expansion using from 30 CRCs. (A) TCR sequencing of tumor tissues from CRC patients (n = 30) enabled UMAP visualization of T cell clonal size, highlighting clonal expansion across different T cell subtypes. **(B)** Histograms showing clonal size in CD8$^+$ Tex (top), CD8$^+$ Tem (middle), and CD8$^+$ Trm (bottom), comparing low and high *CEBPB* expression groups derived from scRNA-seq data of 30 CRCs. Abbreviations: *CEBPB*, CCAAT enhancer binding protein beta; N/A, not applicable; Tem, effector memory T cell; Tex, exhausted T cell; Trm, tissue-resident memory T cell; UMAP, uniform manifold approximation and projection.

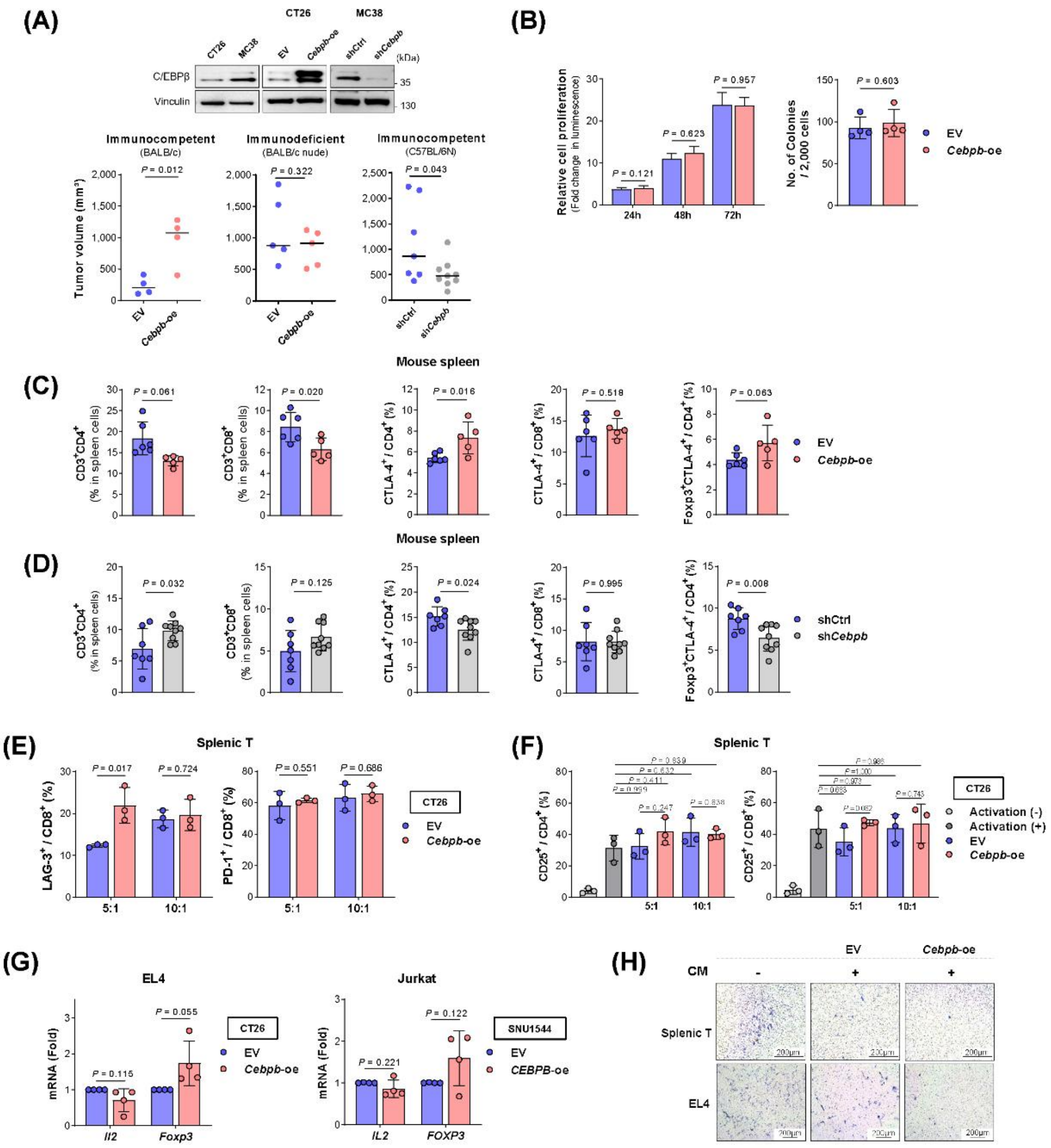

Supplementary Figure S13. Tumor cell C/EBPβ increases CTLA-4 expression in T cells. (A) C/EBPβ levels determined by Western blotting (top); subcutaneous tumor volumes (bottom) after inoculation with EV and *Cebpb*-oe CT26 cells in BALB/c mice (n = 4 each) at day 27 and in BALB/c nude mice (n = 5 each) at day 18, and shCtrl and sh*Cebpb* MC38 cells in C57BL/6N (n = 7 and 9, respectively) at day 18. **(B)** Cell proliferation of EV and *Cebpb*-oe CT26 cells was assessed every 24 h using the CellTiter-Glo assay (right). Colony-forming capacity of EV and *Cebpb*-oe CT26 cells was assessed using a soft agar colony formation assay (left). **(C-D)** Spleen cells were isolated from BALB/c mice injected with EV or *Cebpb*-oe CT26 cells at approximately 21 days post-inoculation **(C)**, and from C57BL/6N mice injected with shCtrl or sh*Cebpb* MC38 cells at 18 days post-inoculation (D). Percentages of

$CD3^+CD4^+$ and $CD3^+CD8^+$ cells among total splenocytes, CTLA-4$^+$ cells within CD4$^+$ and CD8$^+$ populations, and Foxp3$^+$CTLA-4$^+$ cells within the CD4$^+$ population were analyzed by flow cytometry. **(E-F)** Flow cytometry analysis of mouse splenic T cells activated with anti-CD3ε plus anti-CD28 and co-cultured with indicated cells for 72 h, showing LAG-3$^+$ or PD-1$^+$ expression **(E)** and CD25$^+$ expression **(F),** the culture ratio of T cells to CT26 cells is indicated. **(G)** *IL2* and *FOXP3* mRNA levels in EL4 (left) and Jurkat cells (right) co-cultured with EV or *CEBPB*-oe cells, analyzed by real-time PCR. **(H)** Representative images of hematoxylin staining of EL4 and spleen T cells that migrated to the bottom of the transwell insert after 24 h of culture in CM from EV or *Cebpb*-oe CT26 cells.

Abbreviations: C/EBPβ, CCAAT enhancer binding protein beta; CM, conditioned media; CTLA-4, cytotoxic T-lymphocyte associated protein 4; EV, empty vector; Foxp3, forkhead box protein P3; *IL2*, interleukin 2; LAG-3, lymphocyte activating 3; PD-1, programmed cell death protein 1; real-time PCR, real-time polymerase chain reaction; shCtrl, control short hairpin RNA.

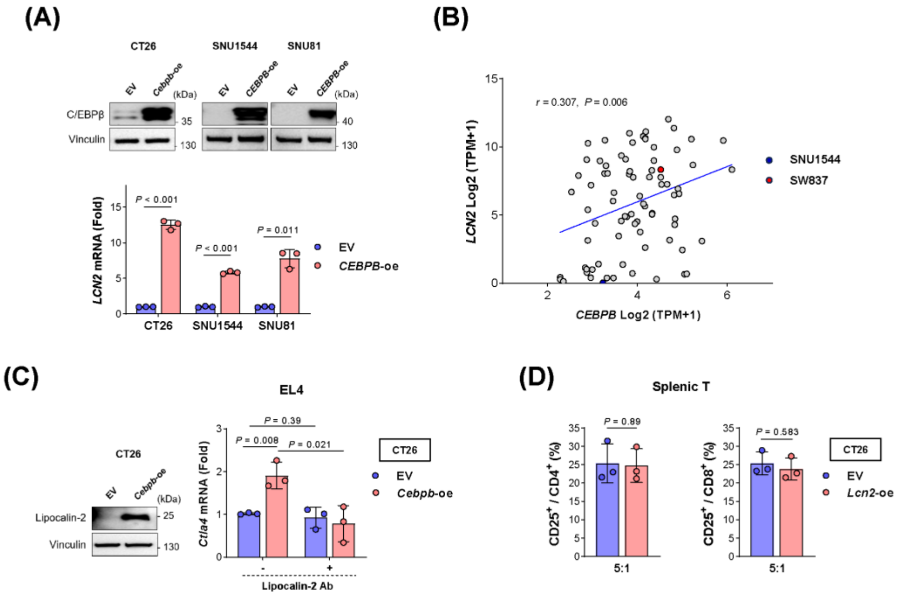

Supplementary Figure S14. Lipocalin-2 mediates CTLA-4 upregulation in T cells. (A) Western blotting analysis confirming C/EBPβ overexpression (top) and real-time PCR analysis of *LCN2* mRNA expression (bottom) in EV and *Cebpb*-oe CT26 cells, and in SNU1544 and SNU81 cells transiently transfected EV or *CEBPB*. **(B)** Correlation between *CEBPB* and *LCN2* mRNA expression among CRC cell lines from the DepMap (Expression public 24Q2) database. **(C)** Lipocalin-2 protein levels in EV and *Cebpb*-oe CT26 cells (left). *Ctla4* mRNA expression in EL4 co-cultured with the indicated cells in the presence of mouse lipocalin-2 neutralizing antibody (1 μg/mL, +) or PBS (-) for 24 h (right). **(D)** Flow cytometry analysis of CD25^{+} cells among CD4^{+} or CD8^{+} cells from mouse splenic T cells co-cultured with EV or *Cebpb*-oe CT26 cells for 48 h at indicated culture ratio of T cells to CT26 cells. The bar graphs represent the mean ± SD; each dot indicates independent biological replicates. All *P* values were calculated using two-tailed Student's *t*-tests. Abbreviations: *CEBPB*, CCAAT enhancer-binding protein beta; CRC, colorectal cancer; *Ctla4*, cytotoxic T-lymphocyte associated protein 4; EV, empty vector; *LCN2*, lipocalin-2; PBS, phosphate-buffered saline; real-time PCR, real-time polymerase chain reaction; SD, standard deviation.
